# Spin polarization induced rapid reconstruction of transition metal oxide for efficient water electrolysis†

**DOI:** 10.1039/d5sc04336k

**Published:** 2025-07-11

**Authors:** Zi-Qiang Chen, Wei-Jie Cai, Hui-Jian Zhang, Kang Xiao, Bolong Huang, Zhao-Qing Liu

**Affiliations:** a School of Chemistry and Chemical Engineering/Institute of Clean Energy and Materials/Guangzhou Key Laboratory for Clean Energy and Materials, Guangzhou University Guangzhou 510006 China lzqgzu@gzhu.edu.cn; b School of Chemistry, South China Normal University Guangzhou 510006 China; c Department of Chemistry, City University of Hong Kong Kowloon Hong Kong China b.h@cityu.edu.hk

## Abstract

Although high-valent metal hydroxyl oxides formed *in situ* through electrochemical oxidation of the metal oxide matrix are key active sites for the oxygen evolution reaction (OER) in transition metal oxides, such a sluggish structural reconstruction largely hinders the electrocatalytic performance. Herein, we present a novel spin polarization engineering strategy to accelerate the formation of high-valent CoOOH, thereby significantly enhancing the OER performance. Through strategic substitutional doping of Mn atoms into the CoO lattice and subsequent confinement of the resulting bimetallic oxides within hollow mesoporous carbon spheres (Mn–CoO/HMCS), the as-prepared catalyst demonstrates markedly enhanced electrocatalytic activity, delivering approximately 5.9-fold higher mass activity compared to the undoped CoO/HMCS counterpart. *In situ* spectroscopy and theoretical calculations elucidate that Mn doping induces lattice distortion and symmetry breaking, which alters the orbital filling of Co with a lower energy barrier for the structural reconstruction from Co^2+^ to Co^3+^. The spin state transition from a high-spin configuration in Co^2+^ to a low-spin state in Co^3+^ further facilitates the formation of CoOOH active intermediates for OER. This work not only paves new avenues for promoting the dynamic reconstruction of active hydroxyl oxides but also highlights the untapped potential of cobalt-based materials through rational electronic structure modulation.

## Abstract 2

The modulation of Co orbital filling lowers the energy barrier for the Co^2+^ to Co^3+^ reconstruction process, facilitating the adsorption/desorption equilibrium of the *OOH in the OER for enhanced efficiency of interfacial electron transfer.

## Graphical abstract

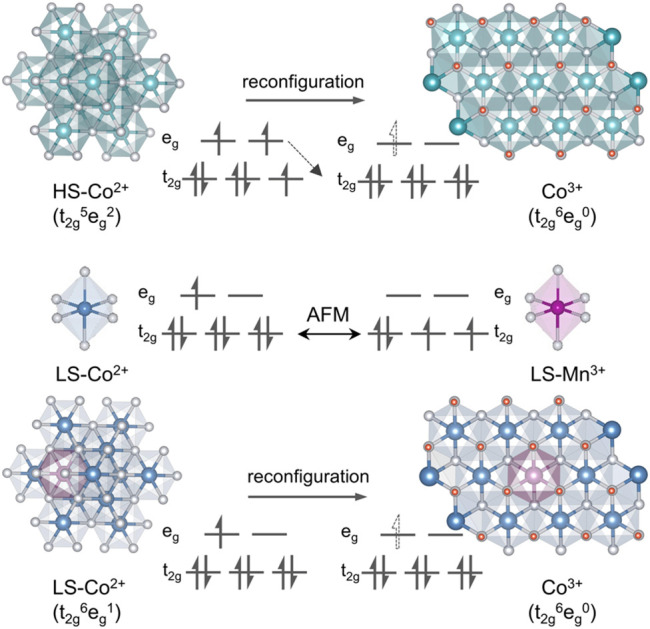

## Introduction

Transition metal oxides (TMOs) have emerged as promising electrocatalysts for the oxygen evolution reaction (OER) due to their low cost, structurally tunable frameworks, and intrinsic OER activity.^1–4^ A growing consensus highlights that TMOs undergo electrochemical reconstruction during OER, evolving into high-valent metal hydroxides (*e.g.*, CoOOH) that serve as the true active species.^5–7^ Among first–row transition metal-based catalysts, cobalt (Co)-containing materials have been intensively investigated, with the high-valent Co state widely recognized as a pivotal determinant of catalytic performance. The CoOOH phase, characterized by its unique 3d electron configuration and strong metal–oxygen covalency, has demonstrated exceptional potential in electrocatalytic water oxidation.^8–12^ In the CoO lattice, octahedrally coordinated Co^2+^ centers (six-fold oxygen coordination in symmetrical octahedral geometry) exhibit stronger M–O covalency than tetrahedral sites, facilitating surface reconstruction. These octahedral metal ions, often exposed on the material surface, act as primary active sites by efficiently interacting with OER reactants.^13–16^ However, the energy-intensive transformation from octahedral CoO to CoOOH requires both Co^2+^ → Co^3+^ oxidation and O–O bond formation remains kinetically sluggish, necessitating high overpotentials and limiting practical applications. Deciphering the surface structural evolution and electronic behavior of CoO during OER is therefore critical for rational catalyst design.

Recent advancements reveal that OER activity is intimately linked to the spin-state-dependent orbital hybridization between metal active sites and adsorbed intermediates, governed by the occupancy of Co 3d-e_g_ orbitals and spin electronic configurations.^17,18^ Elemental doping has emerged as an effective strategy to modulate metal-site electronic structures: by adjusting Fermi-level positions, tuning metal–ligand interactions, and inducing lattice strain, dopants can optimize intermediate adsorption and accelerate reaction kinetics.^19–21^ However, a persistent challenge remains: the instability of reconstructed high-valent metal hydroxides, which often suffer from dissolution of active Co^3+^ species, compromising long-term catalytic durability. Thus, achieving twofold objectives—accelerating the formation of active CoOOH and stabilizing its post-reconstruction structure—represents a key breakthrough direction for OER catalyst development.

In this work, we report a spin-polarization engineering strategy to synergistically modulate Co spin states and promote hydroxyl oxide reconstruction. By encapsulating Mn-substituted CoO quantum dots within hollow mesoporous carbon spheres (Mn–CoO/HMCS), we design a pre-catalyst that enables rapid structural transformation while maintaining stability. Mn doping modulates the occupancy of Co 3d orbitals, inducing a spin-state transition that lowers the energy barrier for CoOOH formation and optimizes the adsorption of oxygen-containing intermediates on active sites. Meanwhile, the nanoconfined microenvironment of mesoporous carbon not only controls the size and morphology of CoO quantum dots, minimizes mass-transfer resistance, but also stabilizes critical CoOOH intermediates, addressing the long-standing issue of reconstruction-induced degradation. This work establishes a dual-function design principle combining electronic structure modulation and nanoconfinement engineering to unlock the full potential of cobalt-based OER catalysts.

## Results and discussion

**Fig. 1:**
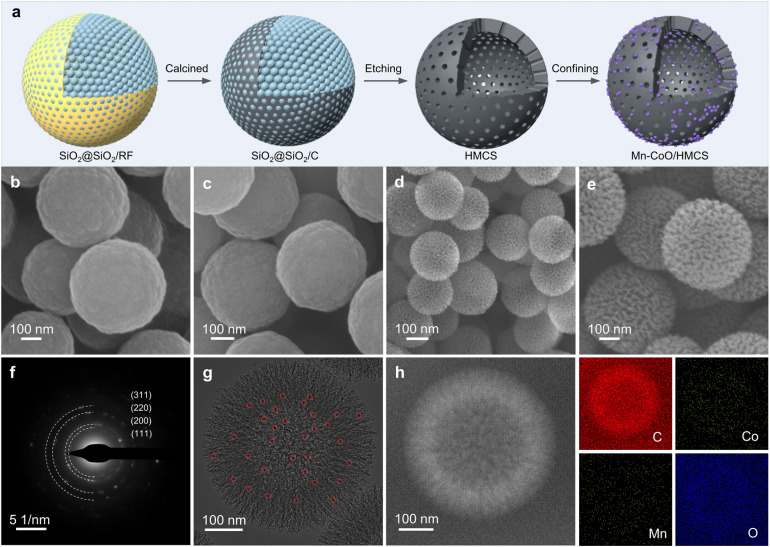
Synthetic process and structural characterization for Mn–CoO/HMCS. (a) Schematic illustration of the synthetic procedure of Mn–CoO/HMCS. (b–e) SEM images of SiO_2_@SiO_2_/RF, SiO_2_@SiO_2_/C, HMCS, and Mn–CoO/HMCS, respectively. (f) The corresponding SAED pattern of Mn–CoO/HMCS. (g) TEM image of Mn–CoO/HMCS. The red circles represent metallic quantum dots. (h) HAADF-STEM image and the corresponding elemental mappings of an individual particle of Mn–CoO/HMCS.

The Mn–CoO/HMCS was synthesized by the hard templating method (Fig. 1a). Initially, resorcinol-formaldehyde (RF) nanoparticles were encapsulated within a SiO_2_ shell, denoted as SiO_2_@SiO_2_/RF (Fig. 1b). Upon carbonization, a core–shell structured SiO_2_(core)@SiO_2_(shell)/C was formed (Fig. 1c). Subsequent etching yielded HMCS (Fig. 1d). Metal ions were then confined within these HMCS through a hydrothermal synthesis process, followed by calcination to form the final Mn–CoO/HMCS composite (Fig. 1e). Scanning electron microscopy (SEM) images reveal that the monodispersed SiO_2_@SiO_2_/C nanocomposite spheres are uniformly dispersed with an average diameter of approximately 400 nm (Fig. S1, ESI†). Transmission electron microscopy (TEM) images display the HMCS post-etching, exhibiting a hollow state with smooth pore channels (Fig. S2, ESI†). This nanoporous structure affords a high surface area and large pore diameter, being 1004 m^2^ g^−1^ and 7.3 nm, respectively (Fig. S3, ESI†). Mn atoms were readily incorporated into the CoO lattice due to their similar size to Co atoms, thereby substituting for the octahedral sites of Co, as evidenced by X-ray diffraction (XRD) results (Fig. S4a and b, ESI†). The diffraction peaks corresponding to CoO shift to lower angles as the increment of Mn content (Fig. S4c, ESI†), indicating that the doping of Mn atoms could not alter the original crystal structure of CoO but merely replace some Co atoms.^22^ Selected area electron diffraction (SAED) patterns only exhibit diffraction rings of cobalt oxide (Fig. 1f), consistent with the results of XRD patterns.^23,24^ In comparison to the pristine HMCS, the synthesized metal oxides are distributed on the surface of the carbon spheres and within the mesoporous channels (Fig. 1g and S5, ESI†), leading to a reduction in pore volume (Fig. S6, ESI†). Energy-dispersive X-ray spectroscopy (EDS) images show the uniform distribution of carbon, cobalt, oxygen, and Mn elements within the carbon spheres (Fig. 1h and S7, ESI†).^25^

**Fig. 2:**
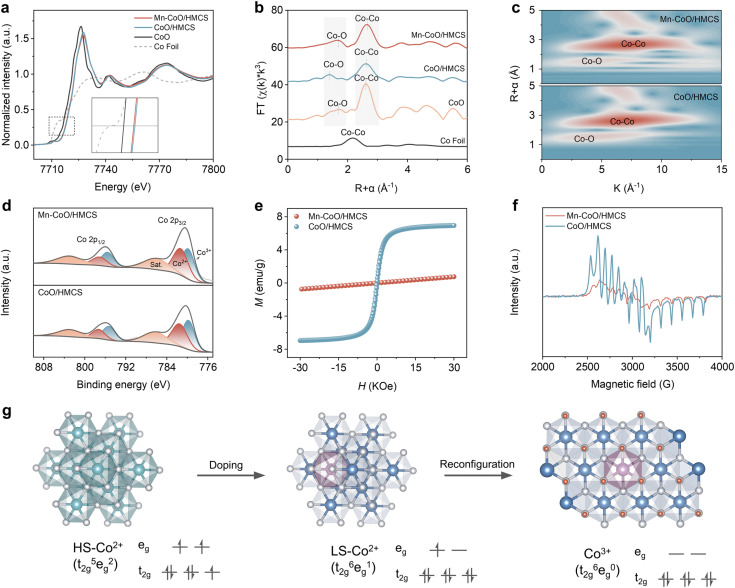
Characterization of the electronic structure for the Mn–CoO/HMCS. (a) XANES spectra at the Co K-edge of Co foil, Mn–CoO/HMCS, CoO/HMCS, and CoO. The inset is the extended image of the selected area. (b) Fourier-transformed Co K-edge EXAFS of Co foil, Mn–CoO/HMCS, CoO/HMCS, and CoO in R spaces. (c) WT-EXAFS contour plots of Mn–CoO/HMCS and CoO/HMCS. (d) X-ray photoelectron spectroscopy (XPS) spectra of Co 3d for Mn–CoO/HMCS and CoO/HMCS. (e) Magnetic hysteresis loop of Mn–CoO/HMCS and CoO/HMCS. (f) EPR spectra of Mn–CoO/HMCS and CoO/HMCS. (g) The crystallographic structure diagram of the material.

To elucidate the impact of Mn on the original CoO material, X-ray absorption spectroscopy (XAS) measurements were conducted to ascertain the changes in the oxidation state of Co. As depicted in Fig. 2a, the absorption energy in the Co K-edge region for Mn–CoO/HMCS is slightly lower than that for CoO/HMCS (inset of Fig. 2a), indicating a reduction in the average oxidation state of cobalt in Mn–CoO/HMCS. In conjunction with the analysis of Mn 3s orbitals (Fig. S8, ESI†), where the average valence state of Mn is +2.8, it can be inferred that the variations in valence states post-doping are likely attributed to the differences in ionic radii between the ions, leading to lattice distortion. This distortion significantly influences the distribution of the electron cloud, resulting in electron transfer from Mn to Co.^26^ The corresponding Fourier-transformed *k3*-weighted extended X-ray absorption fine structure (FT-EXAFS) spectra of Mn–CoO/HMCS at the Co K-edge exhibit two prominent peaks located at 1.65 Å and 2.60 Å, respectively (Fig. 2b and c). According to the EXAFS best-fitting results, the coordination configurations are attributed to the first shell Co–O bonds and the second shell Co–Co bonds (Fig. S9, ESI†). The average Co–O bond distance in the first shell of Mn–CoO/HMCS (1.65 Å) is slightly longer than that of CoO/HMCS (1.39 Å), while the Co–Co bond distance shows no significant changes.^27^ The introduction of Mn doping induces lattice distortion, resulting in variations in Co–O bond lengths, while the Co–Co bond is primarily influenced by the overall structure of the CoO lattice. The incorporation of heteroatoms can modify the original electron distribution, leading to an elevation in electron density within the Co–O bond and consequently increasing its length without altering the initial lattice structure or disrupting the position of the original Co–Co bonds. The expansion of the lattice due to this effect renders the Co–O bond more “relaxed”, which contributes to accelerated reconstruction during the electrochemical process. The Co 2p spectra of Mn–CoO/HMCS shift 0.3 eV towards lower binding energy compared to CoO/HMCS, which is attributed to the electron interaction between Mn atoms and CoO (Fig. 2d). We calculated the ratio of Co^3+^ to Co^2+^, which is an indicator of the surface cobalt oxidation state after Mn ion doping. The results reveal that the Co^3+^/Co^2+^ ratio decreases from 1.24 in CoO/HMCS to 1.02 upon doping. As previously mentioned, the Mn–CoO/HMCS exhibits a lower absorption energy in the cobalt K-edge absorption region compared to CoO/HMCS (Fig. 2a). The O 1 s region is divided into four signals corresponding to the lattice oxygen (O1), chemisorbed oxygen at surface oxygen vacancy (O2), C=O/C–O (O3) and hydroxyl species such as adsorbed water (O4).^12,28^ As the content of Mn elements increases, the proportion of lattice oxygen O1 gradually increases. Mn substitution for Co leads to the formation of higher-valence Mn, which adsorbs additional oxygen atoms, elevating the lattice oxygen ratio. The lattice distortion from doping lengthens Co–O bonds, diminishing oxygen and causing the reduction in cobalt valence state, triggering changes in its spin state (Fig. S10, ESI†). The variation in Co–O bond lengths and Co oxidation states indicates a change in the electronic structure of the material. To further investigate the underlying causes of these changes, we conducted magnetic and EPR tests on the material. Fig. 2e displays the hysteresis loops of Mn–CoO/HMCS and CoO/HMCS at room temperature. CoO/HMCS exhibits a distinct hysteresis phenomenon, even at 300 K, and CoO/HMCS shows a clear hysteresis loop with a coercivity of about 65 Oe (Fig. S11, ESI†). Mn–CoO/HMCS exhibits antiferromagnetic behavior. By comparing the zero-field cooling (ZFC) and field cooling (FC) analysis, CoO/HMCS exhibits ferromagnetic properties with the magnetization intensity decreasing as the temperature rises (Fig. S12a, ESI†).^15,29^ Mn–CoO/HMCS exhibits a pronounced antiferromagnetic transition at *T*_N_ = 11.5 K (Fig. S12b, ESI†). The magnetic variations are intricately linked to the electronic structure of metals and factors such as the incorporation of dopant elements. Electron paramagnetic resonance (EPR) spectroscopy was used to investigate the electron spin configurations of Mn–CoO/HMCS and CoO/HMCS (Fig. 2f). In the EPR test, a strong signal at *g* = 2.28 indicates that CoO/HMCS has more unpaired electron pairs of Co than in Mn–CoO/HMCS. We further analyzed the impact of doping-induced variations in cobalt 3d electron orbital occupancy on magnetic properties through theoretical calculations. The projected density of states (PDOS) of the Co 3d orbitals was analyzed before and after Mn doping. The lower energy *d*_*xy*_, *d*_*yz*_, and *d*_*xz*_ belong to the t_2g_ orbitals, while the higher energy *d*_*z*^2^_ and *d*_*x*^2^−*y*^2^_ may be attributed to the e_g_ orbitals. Clearly, after doping with Mn atoms, the energy of the e_g_ orbitals decreases, with more electrons occupying the t_2g_ orbitals, leading to a change in the spin state (Fig. S13, ESI†).^30^ Based on the magnetic analysis and the filling of unpaired electrons, the orbital distribution of the outermost electrons of Co^2+^ in CoO/HMCS indicates that CoO/HMCS has ferromagnetic properties with a high-spin Co^2+^ state (HS: t_2g_^5^e_g_^2^), where Co is surrounded by six oxygen atoms, forming an octahedral symmetric coordination geometry. Mn doping can affect the Co–O coordination environment and orbital filling, and the spin state changes from high-spin Co^2+^ to low-spin Co^2+^ (LS: t_2g_^6^e_g_^1^) (Fig. 2g). The Mn^2+^ replaces the octahedral position of Co^2+^, and due to the electron transfer between Mn and Co elements during the reaction process, Mn exhibits a +3 valence state and low-spin configuration with a t_2g_^4^e_g_^0^ electron configuration in the MO_6_ non-linear field. According to Goodenough–Kanamori theories, the spin coupling interaction between half-filled t_2g_ orbitals typically exhibits antiferromagnetic characteristics. When considering the higher energy empty e_g_ orbitals, the electron hopping between the half-filled t_2g_ levels and the unfilled e_g_ levels leads to ferromagnetic behavior.^18,31^ The original ferromagnetic CoO/HMCS, caused by the high-spin state of Co^2+^, transitions to antiferromagnetic due to the t_2g_ orbital interaction between Mn and Co (Fig. S14, ESI†). By analyzing the data obtained from the XANES spectra of cobalt, magnetic hysteresis loops, XPS, and EPR, we have gained insights into the modifications of the material electronic configuration and magnetic properties. These changes are ascribed to the lattice distortions and the realignment of electron orbital occupations instigated by the substitution of elements, thereby altering the spin polarization effect.^32^ More specifically, these adjustments have influenced the electronic structure of CoO, ultimately inducing its magnetic character from ferromagnetism to antiferromagnetism.

**Fig. 3:**
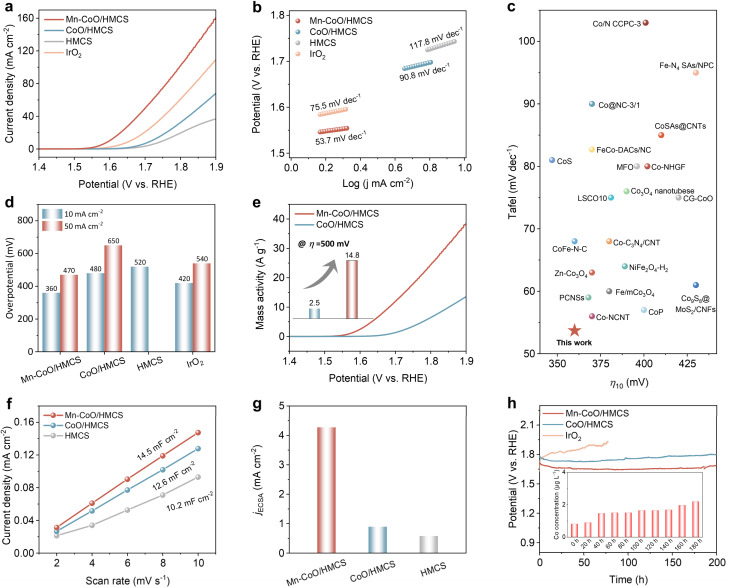
OER performance of Mn–CoO/HMCS and other counterparts in 1.0 M KOH solution. (a) Linear sweep voltammetry (LSV) curves; (b) Tafel plots. (c) OER performance comparison with other previously reported transition metal catalysts. (d) Performance comparison bar chart. (e) Mass activities of Mn–CoO/HMCS and CoO/HMCS. (f) Calculated *C*_dl_ values. (g) The ECSA-normalized current density at an overpotential of 500 mV for Mn–CoO/HMCS, CoO/HMCS, and HMCS, respectively. (h) Chronopotentiometric curves were obtained with the catalytic material on Carbon cloth with constant current densities of 10 mA cm^−2^. The insertion is the change in the amount of Co dissolved in the Mn–CoO/HMCS stability test.

The OER activity was evaluated by LSV in Fig. 3a. The as-optimized Mn–CoO/HMCS shows a higher current response through the entire potential range compared with other catalysts (Fig. S15a, ESI†). Moreover, the Mn–CoO/HMCS has a lower Tafel slope (Fig. 3b, 53.7 mV dec^−1^) than CoO/HMCS (90.8 mV dec^−1^), HMCS (117.8 mV dec^−1^), and IrO_2_ (75.5 mV dec^−1^). The lower Tafel slope indicates more facile OER kinetics on Mn–CoO/HMCS compared to other catalysts (Fig. 3b and S15b, ESI†). As illustrated, under various OER evaluation criteria, the reaction kinetics of Mn–CoO/HMCS is superior to other catalytic materials. Mn–CoO/HMCS also demonstrates superior performance among most of the previously reported transition metal-based OER catalysts (Fig. 3c, d and Table S1 ESI†). The mass activity of Mn–CoO/HMCS is much greater than that of CoO/HMCS. At *η* = 500 mV, the mass activity of Mn–CoO/HMCS was 5.9 times of CoO/HMCS (Fig. 3e and Table S2 ESI†). Turnover frequency (TOF) values were also evaluated, and the results show that Mn–CoO/HMCS affords much higher TOF values at different *η* than the CoO/HMCS electrocatalysts, manifesting high intrinsic activity (Fig. S15c†). The electrochemical surface area was measured by double-layer capacitance (*C*_dl_) testing (Fig. 3f and S16, ESI†) to investigate the density of reliable active sites of the catalytic material. The *C*_dl_ value of Mn–CoO/HMCS is 14.5 mF cm^−2^, much higher than that of CoO/HMCS (12.6 mF cm^−2^) and HMCS (10.2 mF cm^−2^). Notably, the ECSA-normalized current density of Mn–CoO/HMCS reaches 4.45 mA cm^−2^ at an overpotential of 500 mV, approximately 4.5 times that of CoO/HMCS (0.99 mA cm^−2^), indicating that the enhanced intrinsic activities of Mn–CoO gradually (Fig. 3g). Due to the significant differences in magnetism after doping, we further conducted OER tests under an external magnetic field. It was clearly observed that the performance of the ferromagnetic CoO/HMCS effectively improved under an external magnetic field. In contrast, the performance of the antiferromagnetic Mn–CoO/HMCS remained essentially unchanged (Fig. S17, ESI†). The reason is that, in ferromagnetic materials, the atomic magnetic moments spontaneously align parallel to form magnetic domains in the absence of an external magnetic field. When an external magnetic field is applied, these domains gradually align with the direction of the external magnetic field, increasing the material magnetization strength and resulting in a stronger macroscopic magnetism.^33^ The ordered arrangement of magnetic moments enhances the hybridization between the 3d orbitals of metal ions and the 2p orbitals of oxygen, increasing the spin density on oxygen atoms.^34^ Moreover, the external magnetic field strengthens the spin polarization effect of the material, thereby promoting electron transfer. This spin polarization effect can reduce the overpotential of the OER reaction, improving reaction efficiency.^35^ Since the total magnetic moment of antiferromagnetic materials changes little under an external magnetic field, their macroscopic magnetic properties, such as magnetic permeability, remain essentially unchanged. Therefore, their performance does not significantly improve under an external magnetic field like ferromagnetic materials. Remarkably, Mn–CoO/HMCS has excellent durability for 200 h without obvious degradation (Fig. 3h). Inductively coupled plasma optical emission spectroscopy (ICP-MS) was used to quantify the dissolved elements in the electrolyte (Table S3 ESI†), and only a trace amount of cobalt element was dissolved out (10.4 μg L^−1^) along with 200 h of stability testing. Interestingly, a substantial dissolution of Mn occurred (relative to Co), accompanied by the dissolution of oxygen (Fig. S18 and Table S3 ESI†). This results in the formation of oxygen vacancies, thereby causing local charge imbalance on the material surface.^36^ The formation of oxygen vacancies (Fig. S19, ESI†) causes lattice distortion in the material, and this distortion acts as active sites, promoting the adsorption and reaction of reactants, thereby facilitating surface reconstruction.^37^ To restore charge balance, Co^2+^ ions on the material surface are oxidized to Co^3+^ ions, significantly increasing the concentration of surface Co^3+^ ions. XPS analysis revealed (Fig. S20, ESI†) that after testing, the Co element primarily exists in the trivalent state (Co^3+^). This indicates that the oxidation state of Co undergoes significant changes during the oxygen evolution reaction (OER), and these changes are closely related to the formation of oxygen vacancies and lattice reconstruction behavior. OER is one of the most important half-reactions in metal–air batteries. To further explore its material application, we conducted investigations into its ORR properties and assembled it into zinc–air batteries, demonstrating its significant bifunctional activity (Fig. S21–S24, ESI†).

**Fig. 4:**
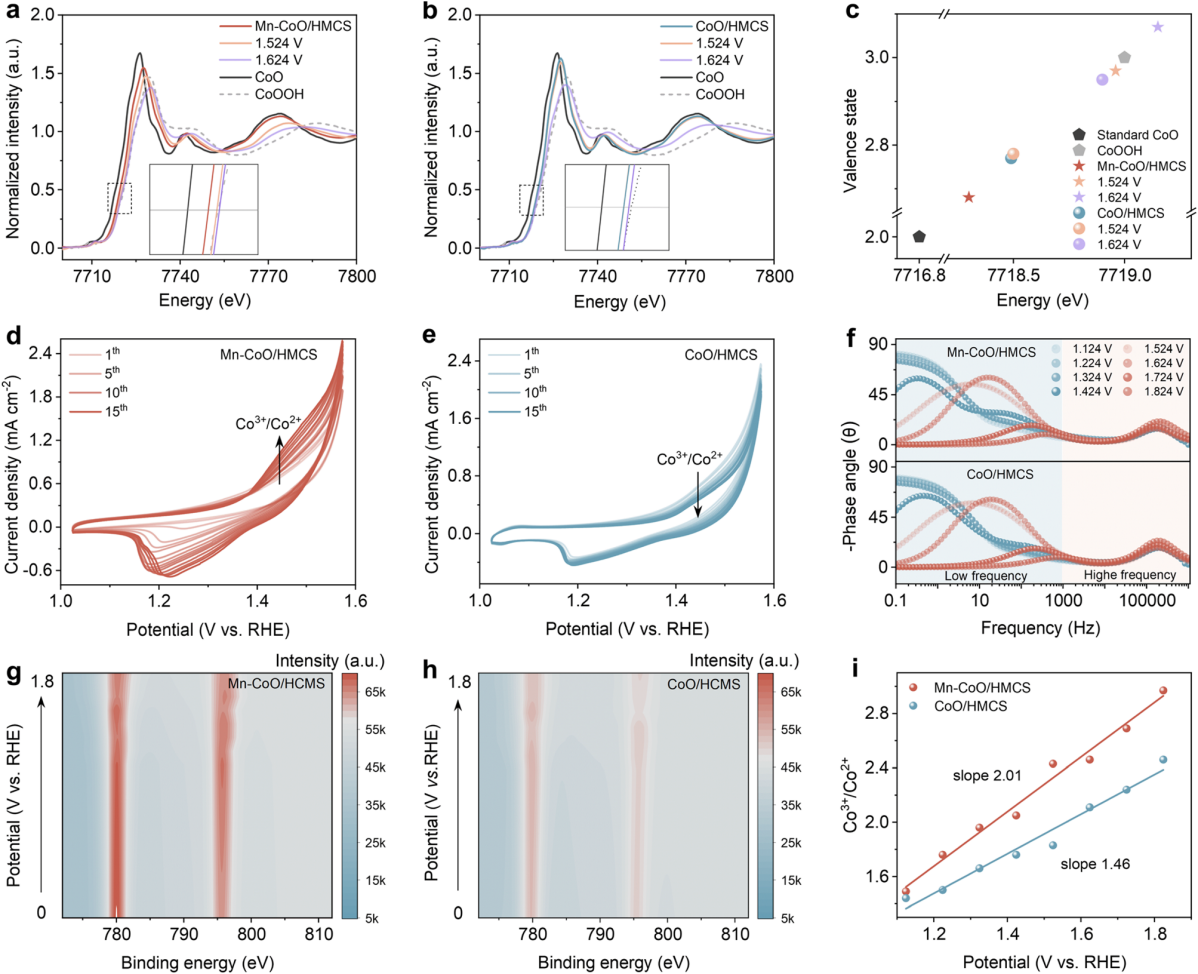
Dynamic reconstruction characterization. Co K-edge XANES spectra of Mn–CoO/HMCS (a) and CoO/HMCS (b) recorded in 1.0 M KOH under pristine state and voltages of 1.524 V and 1.624 V for OER, the standard CoO and CoOOH as references. (c) The calculated relationship between oxidation states and the Co K-edge positions by a half-height method. The selected initial 15-cycle CV of (d) Mn–CoO/HMCS and (e) CoO/HMCS at 0.5 mV s^−1^ in 1 M KOH. (f) Bode plots of the Mn–CoO/HMCS and CoO/HMCS at 1.124–1.824 V potential. Contour plot of Co^3+^ in the *in situ* XPS spectra of Mn–CoO/HMCS electrocatalyst (g) and CoO/HMCS electrocatalyst (h). (i) The variation of the Co^3+^/Co^2+^ redox state in Mn–CoO/HMCS and CoO/HMCS within the potential range from 1.124 V to 1.824 V as analyzed by *in situ* XPS.

We conducted a detailed experimental analysis to investigate the enhanced mechanism of OER activity during cobalt oxide reconfiguration. First, *in situ* electrochemical XANES was performed on Mn–CoO/HMCS and CoO/HMCS to gain a deeper understanding of the rate of valence state changes of the Co element during the OER process (Fig. 4a and b). When the applied voltage is increased to 1.524 V, the Co K-edge peak of Mn–CoO/HMCS shifts to higher energy by 0.65 eV, indicating that cobalt undergoes a peroxidation process triggered by OH adsorption, which leads to a significantly increased valence state. However, the Co K edge of CoO/HMCS has not changed significantly. When the applied voltage is increased to 1.624 V, the Co K-edge peak of Mn–CoO/HMCS has already surpassed that of the standard CoOOH, indicating that cobalt atoms have been obviously oxidized to a high valence state, and some even reaching a +4 oxidation state. In contrast, the Co K-edge of CoO/HMCS only shifts by 0.42 eV, indicating its chemical oxidation state is lower than that of CoOOH (Fig. 4c). According to the fitting results, the coordination configurations after the application of potential were analyzed (Fig. S25 and S26, ESI†),^38^ In the Fourier-transformed k^3^-weighted EXAFS and wavelet transform of the Co K-edge, the Co–O bond length of Mn–CoO/HMCS changed from 1.64 Å to 1.52 Å. When the applied voltage is 1.624 V, with a bond length change of 0.12 Å, while the Co–O of CoO/HMCS only changes from 1.39 Å to 1.48 Å, with a bond length change of 0.09 Å. The aforementioned statement suggests that the structural transformation of Mn–CoO/HMCS is more pronounced and demonstrates a higher rate of reconstruction during the same potential change process (Fig. S27 and S28, ESI†).^39^ The pre-oxidation of Co^2+^ to a higher oxidation state is a key step to generate active CoOOH species for OER. Fig. 4d shows the selected CV curves of Mn–CoO/HMCS for the first 15 cycles. There are an anodic peak and a cathodic peak at 1.5 V and 1.2 V, respectively, which are related to the CoO/CoOOH redox process CoO + OH^−^ → CoOOH + e^−^. The current density of CV increases with the number of cycles, indicating that Mn–CoO/HMCS undergoes initial irreversible surface reconstruction, leading to the conversion of low-valence Co^2+^ to high valence Co^3+^. In contrast, CoO/HMCS exhibits relatively stable changes during the CV process (Fig. 4e), suggesting that Mn-doped CoO is more susceptible to reconstruction and facilitates the generation of Co^3+^. We also performed *in situ* electrochemical impedance spectroscopy (*Operando* EIS) tests under different applied biases. The Bode plots collected on Mn–CoO/HMCS and CoO/HMCS under biases from 1.124 V to 1.824 V are shown in Fig. 4f. By combining the analysis of Bode and Nyquist plots, we can evaluate the rate of the catalyst electro-oxidation process and the kinetic characteristics of the OER.^40–42^ The diameter of the first semicircle corresponds to the high-frequency region, representing the catalyst electro-oxidation. When the applied potential exceeds 1.524 V, two semicircles appear, and the diameter of the second semicircle corresponds to the OER process (Fig. S29, ESI†). Before 1.424 V, the equivalent resistance R1 is relatively large (Fig. S30a, ESI†), indicating weak charge transfer and slow electrocatalytic oxidation. When the applied potential reaches 1.624 V, the phase angle in the high-frequency region of Mn–CoO/HMCS at 1.624 V is not only smaller than that of CoO/HMCS but also lower compared to the phase angle at 1.124 V (Fig. S30b, ESI†).^43^ This phenomenon is reflected in the Nyquist plot as a smaller diameter of the first semicircle, corresponding to a smaller value of the charge transfer resistance R1 in the equivalent circuit, indicating that the electro-oxidation process of Mn–CoO/HMCS is more rapid, and its surface reconstruction rate exceeds that of CoO/HMCS. When the potential reaches 1.624 V, the appearance of OER-related resistance R2 in the equivalent circuit is observed. In the Nyquist plot of Mn–CoO/HMCS, the second semicircle exhibits a decreasing trend with increasing potential, consistently displaying a smaller radius compared to CoO/HMCS. These findings indicate that Mn–CoO/HMCS possesses lower charge transfer resistance and superior kinetic characteristics during the OER process (Fig. S30c and d, ESI†).^44^ The subsequent *in situ* XPS studies were conducted to investigate the surface properties and electronic structure of the material. Comparing the ratio of Co^3+^ to Co^2+^ before and after applying potential provides an indicator of how metal cation doping enhances the rate of surface cobalt oxidation reconstruction (Fig. S31, ESI†). The corresponding Co^3+^ contour map of the XPS spectra demonstrates a higher Co^3+^ content in Mn–CoO/HMCS compared to CoO/HMCS, with more significant changes in the Co^3+^/Co^2+^ ratio as a function of potential (Fig. 4g and h).^45^ This suggests that Mn–CoO/HMCS undergoes faster reconstruction (Fig. 4i).^46,47^ The presence of low-spin Mn facilitates profound remodeling of CoO, thereby enhancing the surface reorganization capability and promoting the formation of the more active CoOOH phase. Consequently, the interaction between low-spin states of Mn and Co not only expedites surface reconstruction but also endows the catalyst with exceptional reaction kinetics owing to its remarkably fast electron transfer ability.

**Fig. 5:**
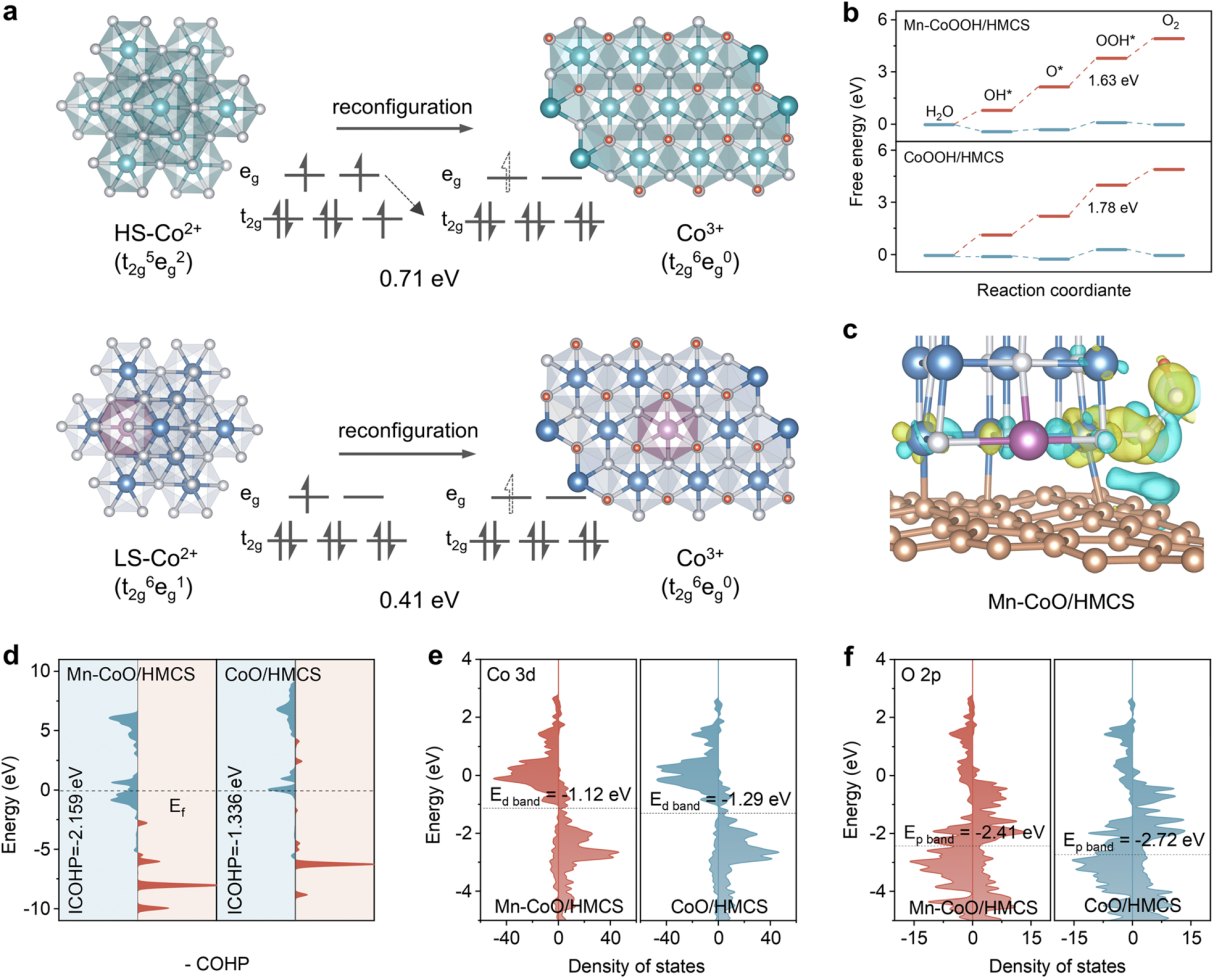
Mechanistic studies of Mn-doped CoO confined within HMCS. (a) Reconstructed structural changes and 3d orbital filling changes of Mn–CoO/HMCS and CoO/HMCS. (b) Gibbs free energy diagrams of Mn–CoOOH/HMCS and CoOOH/HMCS under the bias potential of 0 V and 1.23 V *vs.* reversible hydrogen electrode (RHE). (c) The charge density of *OOH adsorption on the Mn–CoO/HMCS catalyst model. (d) Crystal Orbital Hamilton Population (COHP) analysis of OOH* adsorption on Mn–CoO/HMCS and CoO/HMCS models. (e) PDOS for Co 3d orbital of Mn–CoO/HMCS and CoO/HMCS. (f) PDOS for O 2p orbital of Mn–CoO/HMCS and CoO/HMCS.

The DFT calculation was conducted to elucidate theoretical insights into the surface reconstruction phenomenon (Fig. S32, ESI†). For the CoO model, Co atoms are centered among six oxygen atoms, forming an octahedral symmetrical coordination geometry. A Mn atom replaces the central octahedral Co atom to form the Mn–CoO model. The variable valence characteristics of the metal ions at the octahedral sites are crucial for the multi-electron transfer processes involved in OER, facilitating redox cycles and the reconstruction of metal ions at the octahedral sites, leading to the formation of hydroxo-oxides (MOOH) during the OER process. The oxidation of Co^2+^ to Co^3+^ results in the loss of one 3d electron. The electron loss in the high-spin state of Co^2+^ originates from an e_g_ orbital due to its relatively higher energy level. The transition from high-spin to low-spin state typically involves electron reorganization, requiring an electron from an e_g_ orbital to rearrange to a t_2g_ orbital, which necessitates overcoming a certain energy barrier. However, in the case of Co^2+^ in its low-spin state, only an electron from an e_g_ orbital needs to be lost as the t_2g_ orbitals are already fully occupied (Fig. 5a). Experimental results demonstrate that the electrocatalyst facilitates the reconstruction of CoO to CoOOH, and Mn optimizes this reconstruction behavior. Subsequently, we calculated the energy barriers for pre-catalysts' reconstruction to elucidate the influence of Mn doping. The reconstruction energy barriers for Mn–CoO/HMCS and CoO/HMCS are determined as 0.41 eV and 0.71 eV, respectively, indicating that Mn doping reduces the reconstruction energy barrier, thereby promoting the reconstruction process (Fig. S33, ESI†). To investigate possible electron redistribution, a charge density difference analysis was performed. The incorporation of Mn into CoO/HMCS results in a more pronounced accumulation in the metallic yellow region (area of electron accumulation), exhibiting a significantly larger charge density difference of Δ*Q* = 0.836*e* compared to Δ*Q* = 0.515*e* for CoO/HMCS alone. Enhanced electron accumulation signifies accelerated electron transfer, which facilitates the adsorption of redox and *OH reaction intermediates (Fig. S34, ESI†).^48^ Based on the widely recognized 4e^−^ adsorbate evolution mechanism (*, *OH, *O, and *OOH, where * represents the active sites), the optimized structures of the intermediates in the Gibbs free energy profiles for Mn-doped and non-doped catalysts, both being reconstructed, are determined. We constructed models to explore the OER reaction pathways on the oxidized surfaces of CoO/HMCS, CoOOH/HMCS, Mn–CoO/HMCS, and Mn–CoOOH/HMCS (Fig. S35 and S36, ESI†). For CoO/HMCS, CoOOH/HMCS, Mn–CoO/HMCS, and Mn–CoOOH/HMCS, the rate-determining step (RDS) is O → OOH. Mn–CoOOH/HMCS, Mn–CoO/HMCS, CoOOH/HMCS, and CoO/HMCS have Gibbs free energy values of 1.63, 1.69, 1.78, and 1.89 eV, respectively, for the rate-determining step, indicating that the Mn-doped enhanced surface promotes the adsorption of *OOH (Fig. 5b and S37, ESI†).

To gain a deeper understanding of how the elements affect reconstruction, additional calculations revealed that the introduction of Mn facilitates OOH adsorption onto surface cobalt (Co) atoms, thereby promoting charge transfer within the Co–OOH bond (Fig. 5c and S38, ESI†).^49^ The crystal orbital Hamiltonian population (COHP) analysis was also conducted, where positive and negative values correspond to bonding and anti-bonding contributions, respectively.^50,51^ Through the analysis of the filling of bonding and anti-bonding orbitals, it was observed that the bonding state of the adsorbed intermediate *OOH on Co in Mn–CoO/HMCS is larger than that on CoO/HMCS (Fig. 5d). The formation of CoOOH involves the transformation from Co(OH)_2_ to CoOOH, a process that includes the binding of *OOH. The Mn-doped CoO/HMCS surface enhances the adsorption of *OOH, thereby enhancing the conversion to CoOOH.^52^ It is well known that the higher the central energy of the d band, the lower the proportion of electrons filled in the antibonding orbital, thereby enhancing the metal adsorption (interaction) with oxygen-containing intermediates. Notably, the d-band center of Co and the p-band center of O in Mn–CoO/HMCS are closer to the Fermi level than in CoO/HMCS, which is not only conducive to the exposure of active sites and the adjustment of electronic structure but also related to the strong adsorption of oxygen intermediates. The energy difference between the d-band center of Co and the p-band center of O is reduced to 1.29 electron volts, indicating that the covalency between the 3d orbitals of Co and the 2p orbitals of O in Mn–CoO/HMCS is enhanced, which suggests that the atoms at this site have high reactivity and are prone to further evolve to a high valence configuration during the charging states (Fig. 5e, f and S39, ESI†).^53^ From the theoretical viewpoint, we prove that the construction of the Mn–Co bimetallic center can induce charge redistribution and accelerate surface reconstruction, which is the key to accelerating the oxygen evolution kinetics.

## Conclusions

In summary, we present a simple strategy for accelerating the generation of high-valence metal hydroxyl oxides through doping-induced alteration of orbital occupancy. Experimental results demonstrate that the Mn doping atoms not only transition the Co^2+^ in CoO from a high-spin state to a low-spin state but also elicit antiferromagnetic interactions with Mn. This transformation of the spin state facilitates the conversion of Co^2+^ to Co^3+^, thereby promoting the adsorption/desorption equilibrium of the *OOH reaction intermediate in the OER and enhancing the efficiency of interfacial electron transfer. Furthermore, dynamic experimental observations reveal that Mn-doped CoO can generate CoOOH more rapidly than its undoped counterpart, significantly enhancing the catalytic performance of OER. Concurrently, the confinement effect of the HMCS substantially boosts the activity and stability of the material in the OER. These findings not only offer a new perspective for the design and preparation of high-performance electrocatalysts but also lay the groundwork for a deeper understanding of the intrinsic activity of metal oxides in the electrocatalytic process.

## Author contributions

Z.-Q. L. and K. X. proposed the idea. Z. C. designed the experiments and performed the materials synthesis and physical characterizations. Z. C. carried out the electrochemical experiments. Z. C, K. X., B.-l. H. and Z.-Q. L. analyzed and discussed all experimental results and drafted the manuscript. All authors checked the manuscript and agreed with the content.

## Conflicts of interest

There are no conflicts to declare.

## Supplementary Material

SC-016-D5SC04336K-s001

## Data Availability

The data supporting this article have been included as part of the ESI.†
