# Supplementary material for: Spin polarization induced rapid reconstruction of transition metal oxide for efficient water electrolysis
Source: Chem Sci. 2025 Jul 11;16(32):14750–9. doi: 10.1039/d5sc04336k (PMC12265186; doi:10.1039/d5sc04336k)
Supplement: SC-016-D5SC04336K-s001 [file SC-016-D5SC04336K-s001.pdf]

Supplementary Information (SI) for Chemical Science.
This journal is © The Royal Society of Chemistry 2025

Supporting information

Spin Polarization Induced Rapid Reconstruction of Transition Metal Oxide for Efficient Water Electrolysis

Zi-Qiang Chen,[a] Wei-Jie Cai,[a] Hui-Jian Zhang,[a] Kang Xiao,[a,] Bolong Huang,[c,*] and Zhao-Qing Liu[a,b*]

[a] School of Chemistry and Chemical Engineering/Institute of Clean Energy and Materials/Guangzhou Key Laboratory for Clean Energy and Materials, Guangzhou University, Guangzhou 510006, China.

E-mail: lzqgzu@gzhu.edu.cn (Z. Q. Liu)

[b] School of Chemistry, South China Normal University, Guangzhou 510006, China.

[c] Department of Chemistry, City University of Hong Kong, Kowloon, Hong Kong, China.

E-mail: b.h@cityu.edu.hk (B. L. Huang)

1. Materials

All reagents used in this work were of analytical grade and were used without further purification. $C_{16}H_{36}O_4Si$ (99%, AR), $C_6H_4(OH)_2$ (99%, AR), CH_4O (99%, AR), $Co(NO_3)_2 \cdot 6H_2O$ (99%, AR), $Mn(NO_3)_2$ (50%), C_2H_5OH (99%, AR) were obtained Shanghai Macklin Biochemical Co. Ltd. The Carbon cloth (CC) was purchased from Suzhou Sinerole chnology Co.Ltd. and the model is WOS1011.

2. Preparation of electrocatalysts

2.1 Preparation of HMCSs

$SiO_2@SiO_2$/RF nanospheres were prepared according to a modified method[1]. We replace the commonly used tetraethoxy orthosilicate (TEOS) with tetrapropyl orthosilicate (TPOS) as the silica source in view of its slower hydrolysis and condensation behavior, which allows better control during the synthesis. Typically, 70 mL of ethanol, 10 mL of deionized water and 3.0 mL of ammonium hydroxide were stirred together for 10 min at room temperature, and then 3.5 mL of TPOS was added into the solution under stirring. After 15 min, 2.0 mL of freshly prepared ethanol solution of resorcinol (0.4 g) and 0.56 mL of formaldehyde (37 wt%) were added into the solution. After stirring at room temperature for 24 h, the $SiO_2@SiO_2$/RF nanospheres were collected by centrifugation, washed with ethanol for several times, and then dried at 60 °C under vacuum overnight. Then, $SiO_2@SiO_2$/RF was placed in a porcelain boat. After annealing at 700 °C (2 °C/ min) for 4 h in N_2 atmosphere, $SiO_2@SiO_2$/C was obtained. After removing the SiO_2 template with 5 M NaOH for 12 h at 50 °C, the hollow mesoporous carbon spheres (HMCSs) were collected by centrifugation, washed with deionized water and ethanol for several times, and then dried at 60 °C under vacuum overnight for further use. The pore size of HMCSs could be tuned by adjusting the ratios of TEOS/TPOS or ethanol/water. HMCSs with pore size of 2 nm and 10 nm were synthesized with TEOS/TPOS of 4/1 and ethanol/water of 60/20, respectively, while other conditions remain unchanged.

2.2 Preparation of CoO/HMCS

The synthesized hollow mesoporous carbon spheres (HMCS) were put into A 100 ml round-bottomed flask, 1.0 g of urea was added, and then 50 ml of deionized water was added, and ultrasonic treatment was carried out for 10 min to prepare solution A. 72.5 mg $Co(NO_3)_2 \cdot 6H_2O$ was put into a 2.0 mL centrifuge tube and added with 1ml deionized water for ultrasonic treatment for 10 min to prepare solution B. Solution B was transferred to solution A using a

pipette and the mixture was then placed in an oil bath at 85 °C for 6 h. After the oil bath reaction, centrifugal separation, washing, and drying were performed. The resulting solid was then ground and placed in a porcelain boat, annealed at 500 °C (2 °C min^{-1}) for 3 h in N_2 atmosphere.

2.3 Preparation of Mn-CoO/HMCS

Mn-CoO/HMCS was synthesized using the same method as CoO/HMCS, with the difference being the incorporation of 50 wt.% $Mn(NO_3)_2$ into the preparation of Solution B. 50 wt.% $Mn(NO_3)_2$ solutions of 7 μL, 14.5 μL, 29 μL, 58.5 μL and 117 μL were added to B solution. These different additions correspond to the prepared samples Mn-CoO/HMCS-1, Mn-CoO/HMCS-2, Mn-CoO/HMCS, Mn-CoO/HMCS-3 and Mn-CoO/HMCS-4.

3. Electrochemical measurements

Electrochemical measurements were conducted in a standard three-electrode system using the PMC CH808A and CHI 760E electrochemical workstations at room temperature. A mercury/mercury oxide electrode served as the reference electrode, a carbon rod electrode as the counter electrode, and a glassy carbon rotating ring-disk electrode (RRDE, 5 mm in diameter) as the working electrode. Prior to the electrochemical measurements, a catalyst ink was prepared. Specifically, 5.0 milligrams of the catalyst were placed in a mixture of 300 microliters of deionized water, 700 microliters of ethanol, and 20 microliters of Nafion solution, followed by ultrasonication for 30 minutes. Subsequently, 10 microliters of the catalyst ink onto the RRDE, and evaluate the oxygen evolution reaction (OER) and the oxygen reduction reaction (ORR) at a rotation speed of 1600 revolutions per minute in 1 M KOH (pH=13.8) using linear sweep voltammetry (LSV). Before the OER measurement, continuous CV at a scan rate of 50 mV s^{-1} from 1.124 to 1.924 V vs RHE was conducted until a reproducible CV was obtained, which turned out to be around 20 cycles. The OER activity of catalysts was evaluated by linear sweep voltammetry (LSV) curves at a scan rate of 10 mV s^{-1} between 1.124 and 1.924 V vs. RHE. All measured potentials were converted to the RHE scale following the Nernst equation:

$$E_{\mathrm{RHE}} = E_{\mathrm{Hg/HgO}} + 0.0591 \times \mathrm{pH} + 0.098$$

where $E_{\mathrm{Hg/HgO}}$ was the potential directly measured with the Hg/HgO reference electrode. The number of electrons transferred (n) during the ORR and the H_2O_2 yield (%) were determined using the following equations based on the RRDE test results.

$$n = \frac{4I_d}{I_d + \left(\frac{I_R}{N}\right)}$$

$$H_2O_2\% = \frac{200\left(\frac{I_d}{N}\right)}{I_d + \left(\frac{I_R}{N}\right)}$$

where I_d, I_R, and N indicate the disk electrode current, ring electrode current, and collection efficiency of RRDE (34%), respectively. Tafel slopes were calculated by Tafel curves, which were plotted using the equation:

$$\eta = a + b \log|j|$$

where η is the overpotential, j is the current density, a is the intercept and b is the Tafel slope. chronoamperometry (CA), the number of electron transfers (n), and the yield of hydrogen peroxide (H_2O_2%). For the OER stability test, where the metal content in the solution was analyzed, a platinum electrode was used as the counter electrode, the mercury/mercury oxide electrode as the reference electrode, and the working electrode was composed of a metal-catalyst-loaded carbon cloth held by a platinum electrode clip. In situ electrochemical impedance spectra (EIS), were recorded at 1.124 V–1.824 V vs. RHE over the frequency range from 1000 kHz to 0.1 Hz with a 5 mV amplitude. The electrochemically active surface area (ECSA) was measured by the double layer capacitance (C_{dl}) method. The potential difference in the non-Faraday interval is 0.1 V, CV curves at different scan rates (2, 4, 6, 8, 10 mV s^{-1}), This capacitance was calculated by dividing the double-layer charging current by the scan rate (v), as illustrated by the linear relationship between the current (i_c) and, where the slope of the plot corresponds to C_{dl}:

$$C_{dl} = \frac{i_c}{v}$$

To assess the intrinsic activity, TOFs were calculated by the following equation:

$$\text{TOF} = \frac{j \times A}{4 \times n \times F}$$

where j is the current density, A is the geometric area of the electrode, F is the Faraday constant (96,485 C mol^{-1}), and n is the number of active sites. The Co atom is considered to be the active site, calculated based on the content of the element Co measured by ICP (Table S1).

4. Zinc-air battery (ZAB) tests

The rechargeable ZABs test was conducted using homebuilt electrochemical cells. A CHI 760D (CH Instruments, Inc., Shanghai, China) electrochemical workstation was used to collect the test data. Concretely, Zinc foil was used as the metal anode, and the air electrode was fabricated using a Nafion-coated carbon fiber paper loaded with 3.0 mg Pt/C and 3.0 mg catalyst or 3.0 mg Pt/C and 3.0 mg IrO_2 as the air electrode, and a 6.0 M KOH solution was used as the electrolyte.

5. Materials Characterization

The morphology was characterized using a Scanning Electron Microscope (SEM), model JEOL JSM-7600F, in conjunction with a Transmission Electron Microscope (TEM), model FEI-Tecnai G2 F30. X-ray diffraction (XRD) patterns were collected on a Panalytical X'Pert Pro X-ray Powder Diffractometer with Cu Kα radiation at a voltage of 40 kV. Raman spectra and in-situ Raman spectroscopy were recorded on a LabRAM HR Evolution Raman spectrometer (HORIBA Jobin Yvon) using a 532 nm laser as the excitation source. N_2 adsorption-desorption isotherms and pore size distributions were analyzed at 77 K with an ASAP 2460 Version 3.01 surface area and pore size analyzer. The specific surface area of the samples was calculated using the multi-point Brunauer-Emmett-Teller (BET) method, while the pore volume and size distribution were determined using the Barrett-Joyner-Halenda (BJH) method based on the adsorption isotherm. In-situ X-ray Photoelectron Spectroscopy (XPS) was performed on a Thermo SCIENTIFIC ESCALAB 250Xi spectrometer, calibrated with the C 1s peak at 284.8 eV. For in situ XPS, the three-electrode system was first assembled and immersed in 1M KOH solution, allowing stabilization for a brief period (this step was performed rapidly to minimize chemical reaction with KOH), followed by XPS spectral acquisition of the pristine sample without applied voltage. Subsequently, an i-t curve was recorded under a constant 0.2V potential for 1 minute, ensuring current stabilization and uniform bubble generation, after which the sample underwent vacuum transfer and immediate XPS scanning. This procedure was repeated by incrementally applying 0.3V, 0.4V, and so forth up to 0.9V, maintaining each potential for 1 minute with stable conditions before testing and data recording. This approach involves post-reaction vacuum transfer for subsequent XPS analysis, whereas true in-situ characterization would require XPS measurement during the electrochemical process, with immediate transfer to the analysis chamber upon reaction termination. Elemental content loss during the stability test was determined by Inductively Coupled Plasma Atomic Emission Spectroscopy (ICP-AES) using an Agilent 7700 (MS) instrument. X-ray Absorption Spectroscopy (XAS) at the Co K-edge for the powder samples without applied voltage was collected in transmission mode at the Shanghai Synchrotron Radiation Facility (SSRF) in

China. For the samples under bias, XAS was collected in fluorescence mode after applying potential to the samples coated on carbon cloth.

6. Theoretical calculations

The first-principles[2, 3] has been utilized to carry out density functional theory (DFT) calculations within the generalized gradient approximation (GGA) using the Perdew-Burke-Ernzerhof (PBE)[4] formulation. The projected augmented wave (PAW)[5, 6] potentials have been selected to describe the ionic cores, with valence electrons being taken into account using a plane wave basis set with a kinetic energy cutoff of 450 eV. Partial occupancies of the Kohn−Sham orbitals were allowed using the Gaussian smearing method and a width of 0.05 eV. The electronic energy was considered self-consistent when the energy change was smaller than 10^{-6} eV. A geometry optimization was considered convergent when the energy change was smaller than 0.03 eV $Å^{-1}$. In addition, for the Co and Mn atoms, the U schemes need to be applied, and the U has been set as 4.025 and 3.929 eV. The vacuum spacing in a direction perpendicular to the plane of the structure is 20 Å for the surface. The Brillouin zone integration is performed using 2×2×1 Monkhorst-Pack k-point sampling for a structure. Finally, the adsorption energies (E_{ads}) were calculated as $E_{ads} = E_{ad/sub} - E_{ad} - E_{sub}$, where $E_{ad/sub}$, E_{ad}, and E_{sub} are the total energies of the optimized adsorbate/substrate system, the adsorbate in the structure, and the clean substrate, respectively. The free energy was calculated using the equation:.

$$G = E + ZPE - TS$$

where G, E, ZPE and TS are the free energy, total energy from DFT calculations, zero point energy, and entropic contributions, respectively

Supplementary Figures

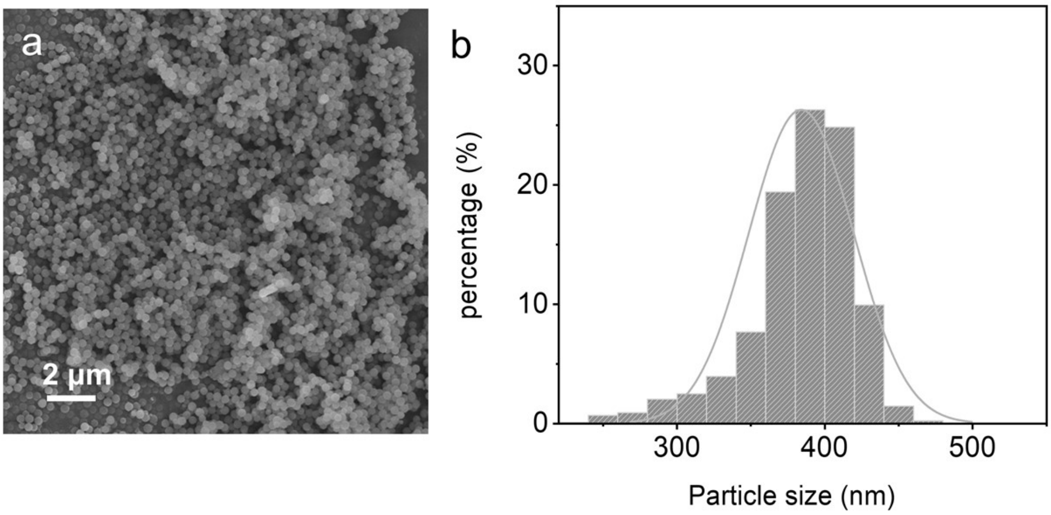

Figure S1. (a) SEM images of SiO_2@SiO_2/C, (b) Size distribution of SiO_2@SiO_2/C.

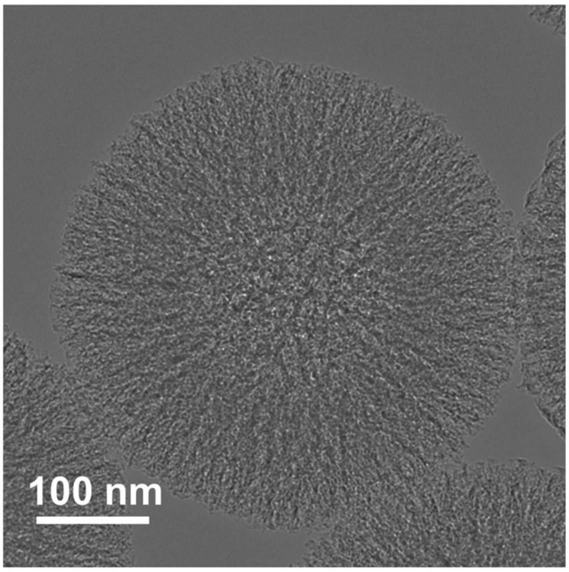

Figure S2. TEM images of HMCS.

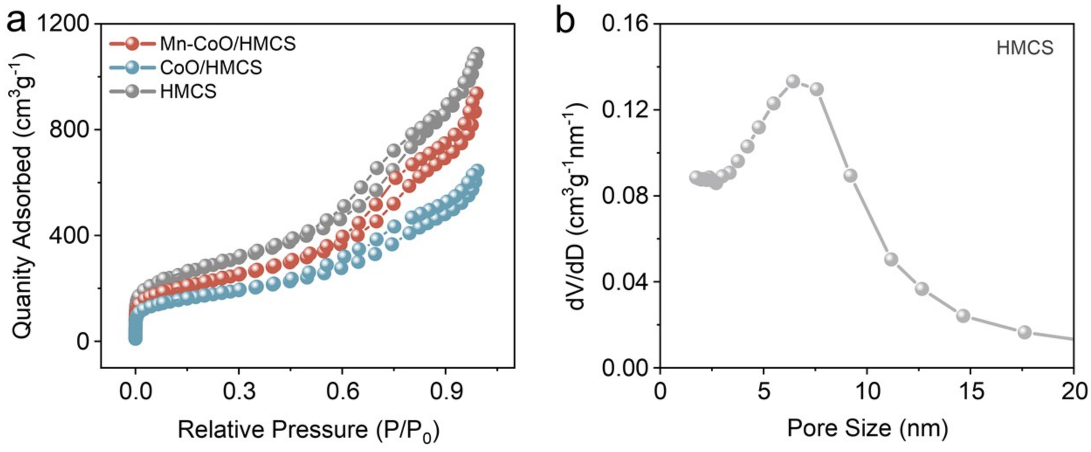

Figure S3. (a) Nitrogen adsorption-desorption isotherms of Mn-CoO/HMCS, CoO/HMCS, and HMCS. (b) BJH Pore Size Distribution of HMCS.

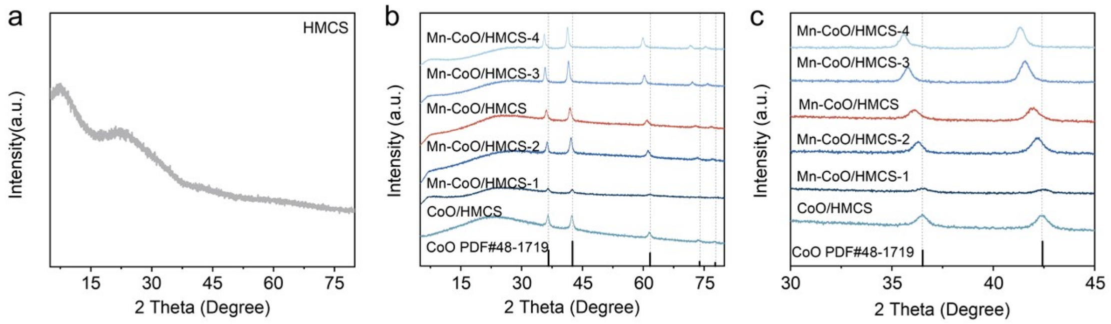

Figure S4. (a) XRD patterns of HMCS. (b) XRD patterns of all catalysts. (c) Partial magnification XRD patterns of all catalysts.

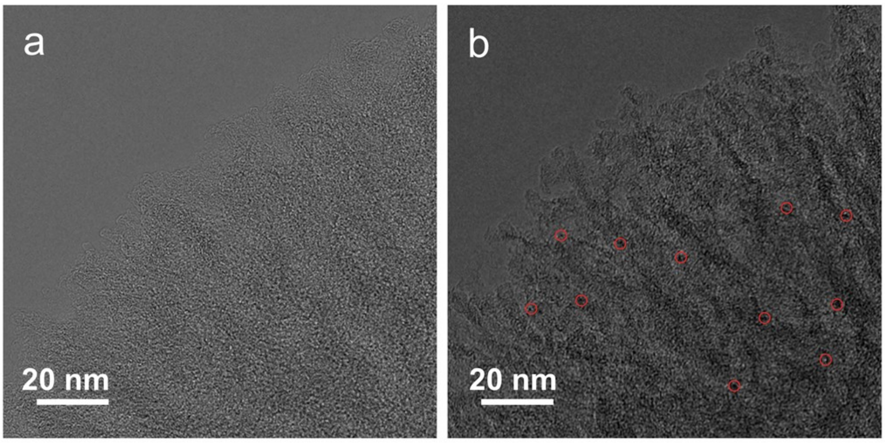

Figure S5. TEM images of (a) HMCS and (b) Mn-CoO/HMCS.

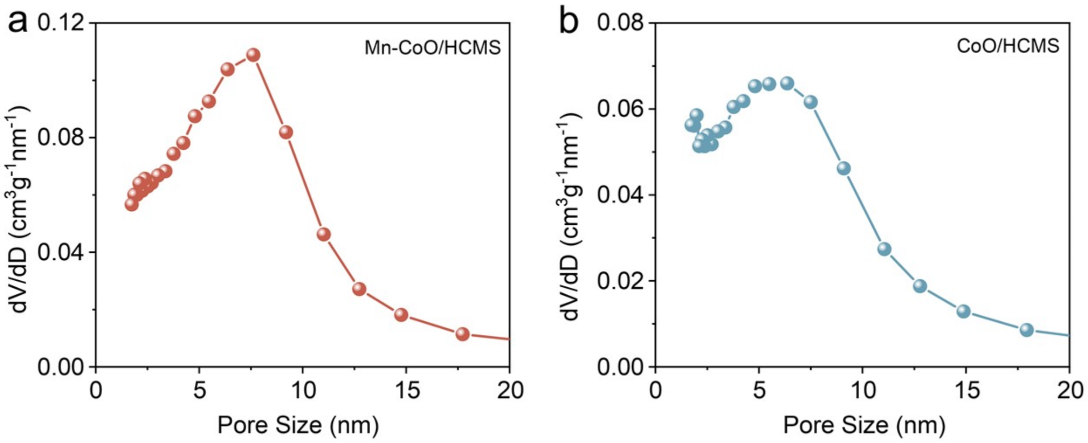

Figure S6. BJH Pore Size Distribution of (a) Mn-CoO/HMCS and (b) CoO/HMCS.

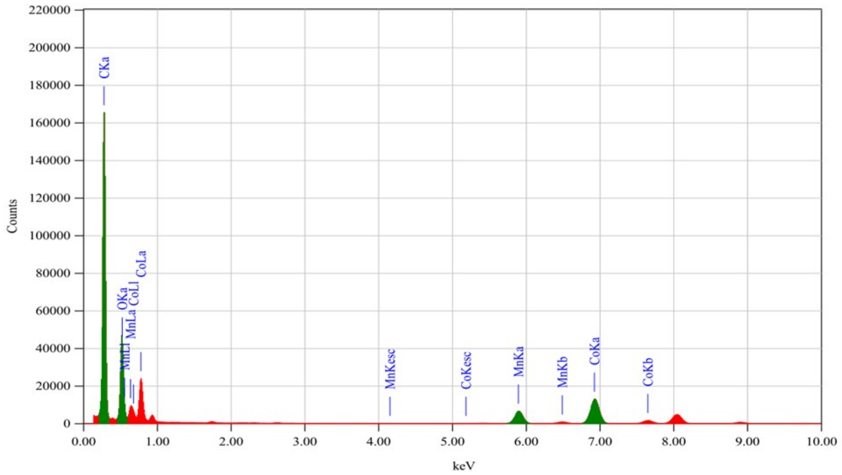

Figure S7. The corresponding EDS of Mn-CoO/HMCS.

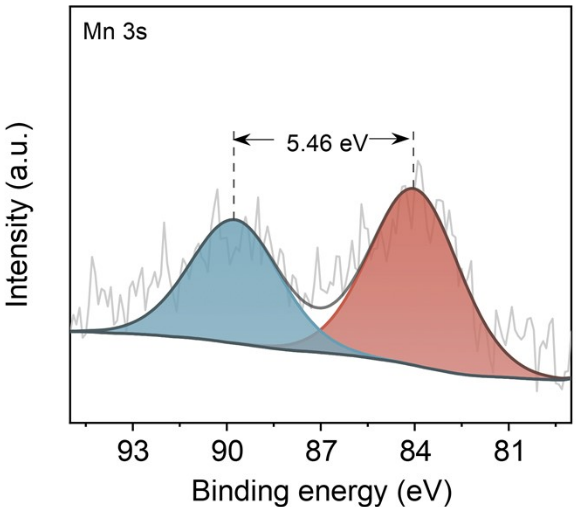

Figure S8. High resolution XPS spectra of Mn 3s for Mn-CoO/HMCS.

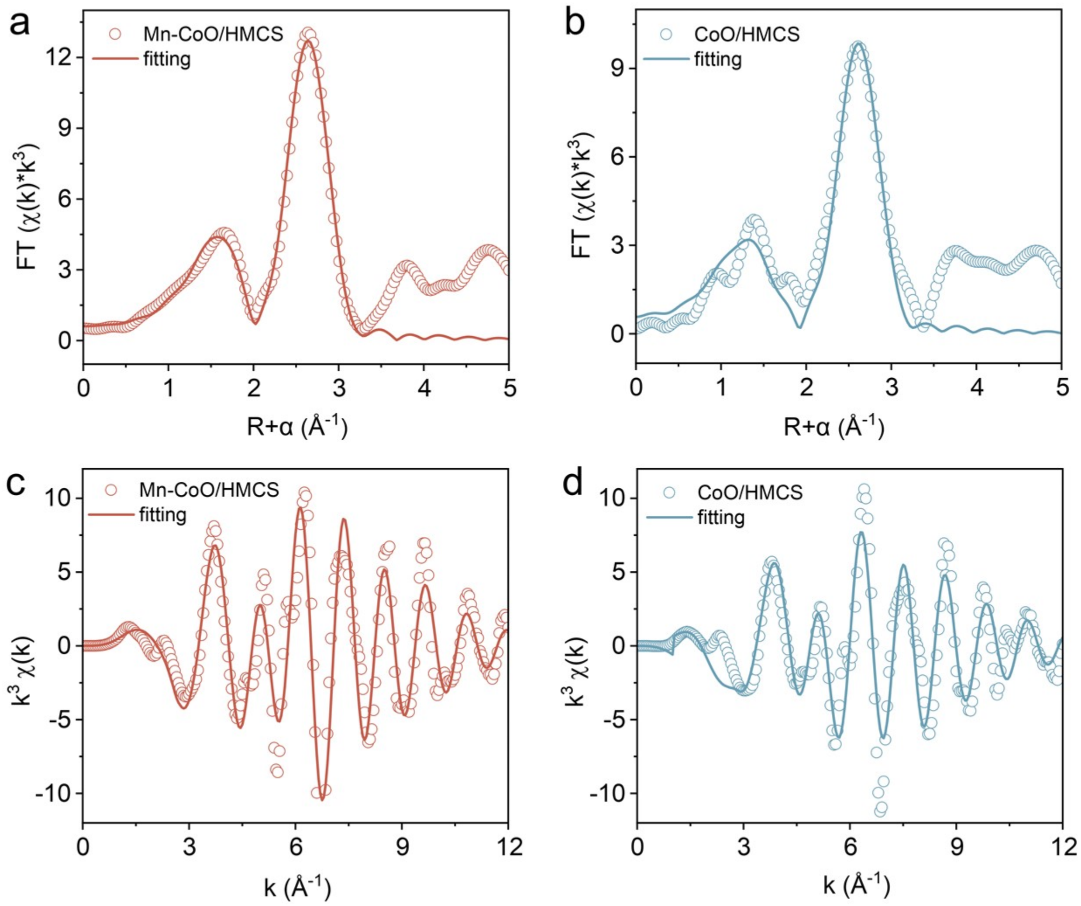

Figure S9. FT-EXAFS fitting results of (a) Mn-CoO/HMCS and (b) CoO/HMCS. EXAFS fitting curves at K space of Co K-edge for (c) Mn-CoO/HMCS and (d) CoO/HMCS.

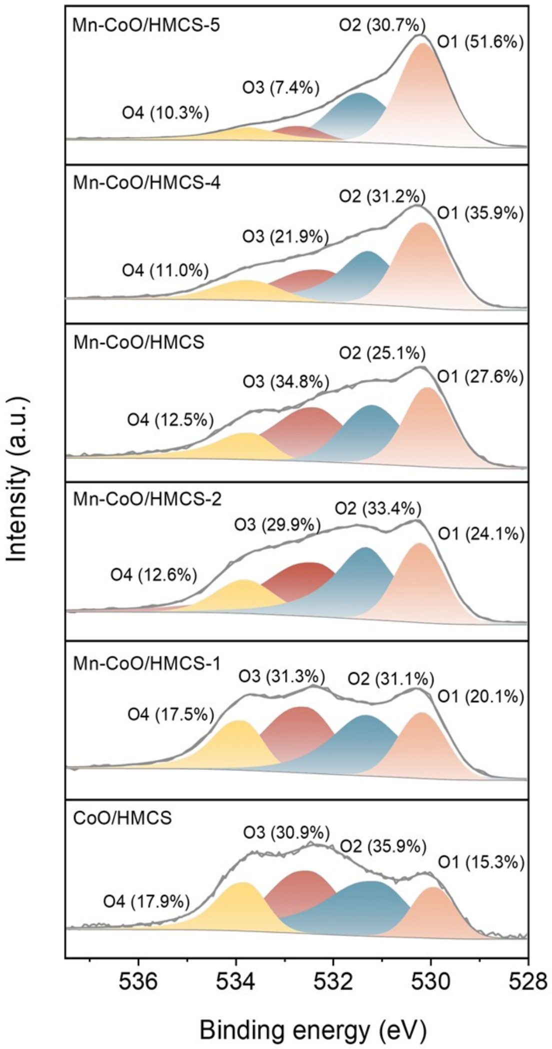

Figure S10. High-resolution O 2p XPS spectra of all catalysts.

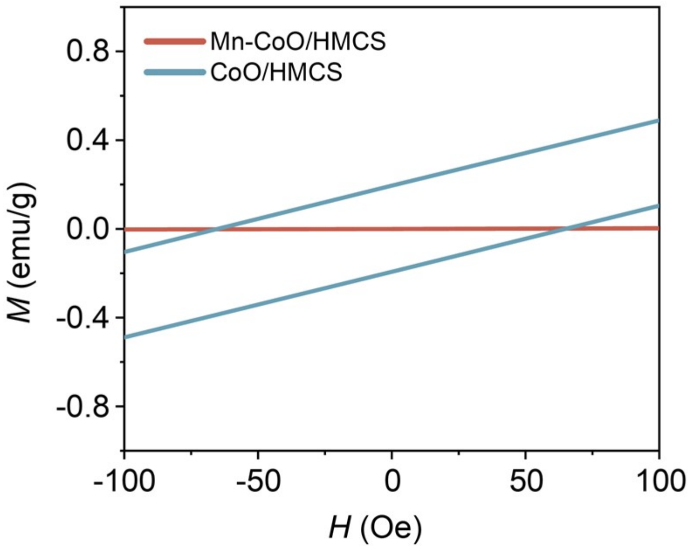

Figure S11. The localized magnified Magnetic hysteresis loop of Mn-CoO/HMCS and CoO/HMCS.

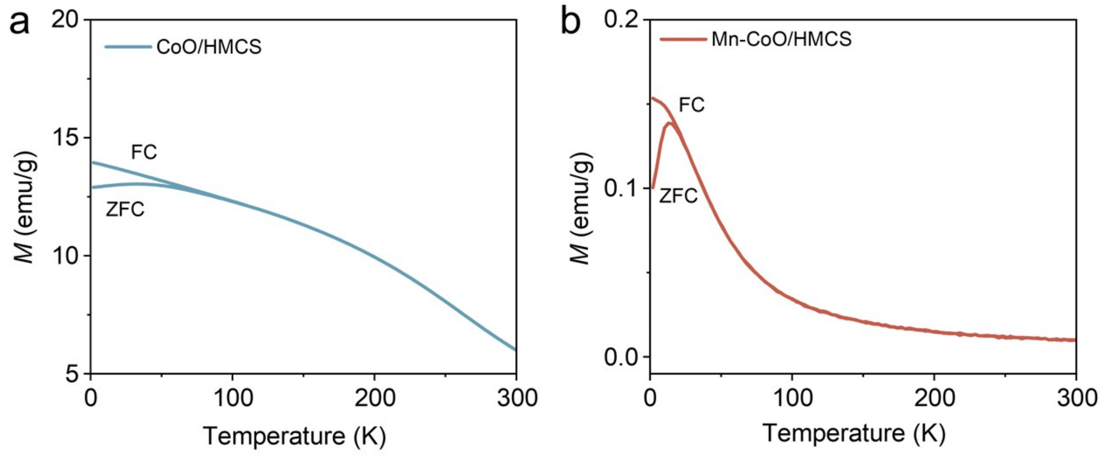

Figure S12. ZFC and FC magnetic results of (a) CoO/HMCS and (b) Mn-CoO/HMCS.

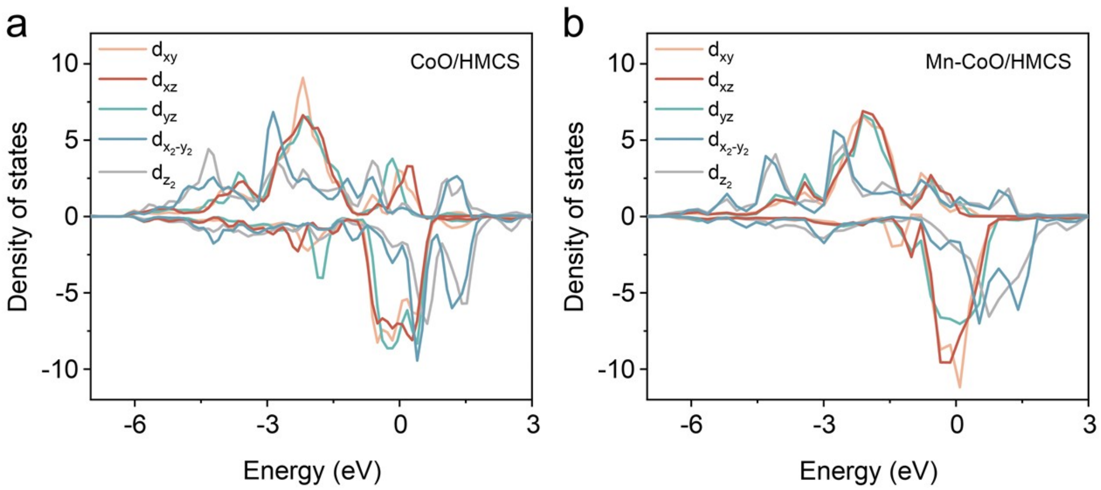

Figure S13. Projected Co 3d orbital density of states of (a) CoO/HMCS and (b) Mn-CoO/HMCS.

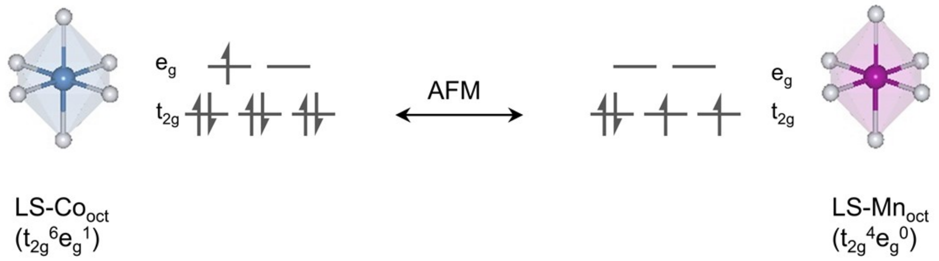

Figure S14. Schematic of the ferromagnetic exchange between Co^{2+} and Mn^{3+}.

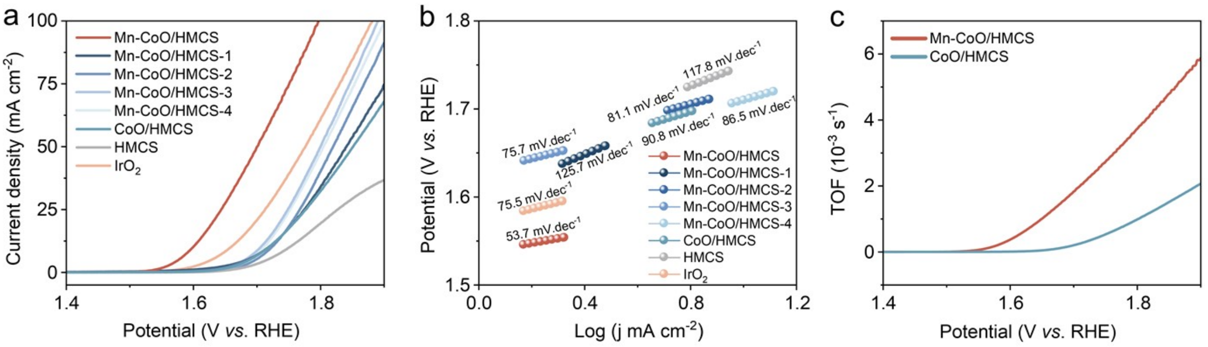

Figure S15. OER performance: (a) LSV curves of all catalysts in 1 M KOH, (b) Tafel plots, (c) TOF curves derived from LSV curves.

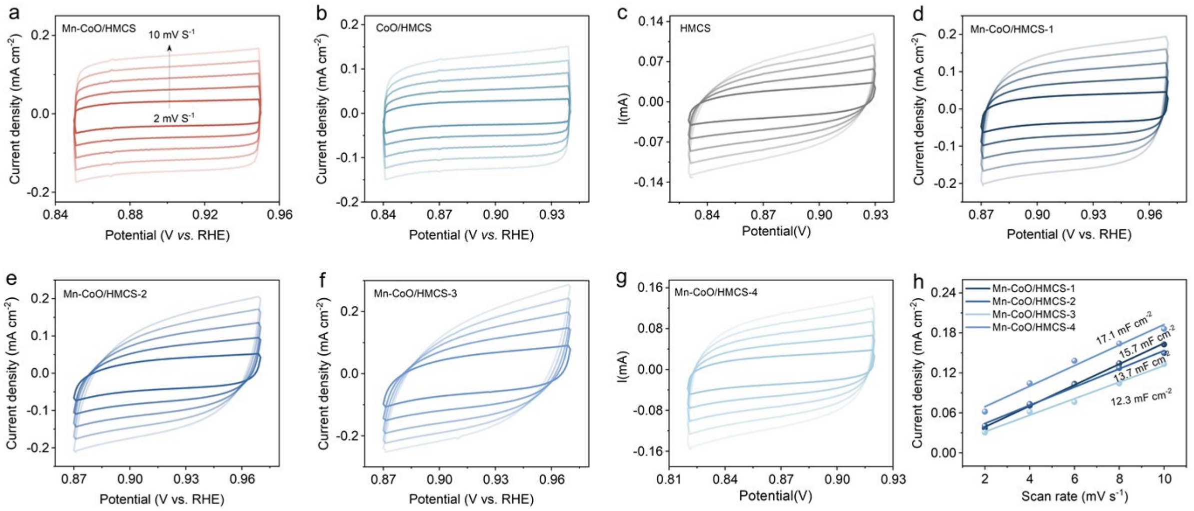

Figure S16. CV curves at different scan rates for (a) Mn-CoO/HMCS, (b) CoO/HMCS, (c) HMCS, (d) Mn-CoO/HMCS-1, (e) Mn-CoO/HMCS-2, (f) Mn-CoO/HMCS-3, and (g) Mn-CoO/HMCS-4. (h) C_{dl} plots of Mn-CoO/HMCS-1, Mn-CoO/HMCS-2, Mn-CoO/HMCS-3 and Mn-CoO/HMCS-4.

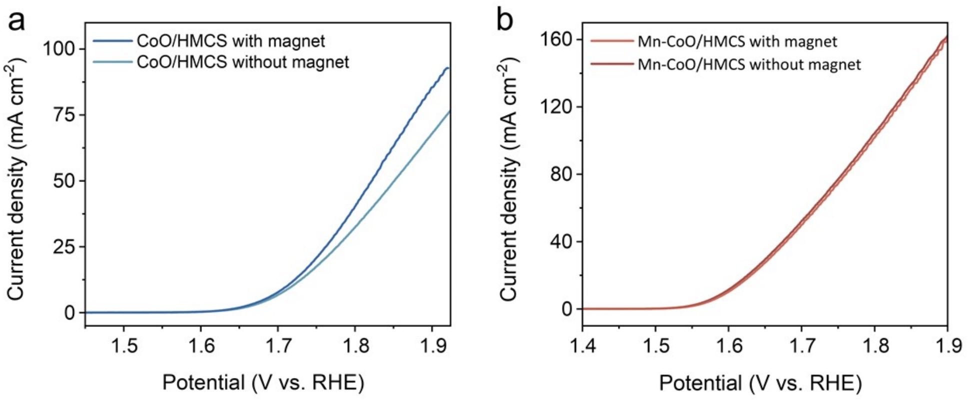

Figure S17. The LSV curves of (a) CoO/HMCS and (b) Mn-CoO/HMCS with and without adding magnetic field (210 mT).

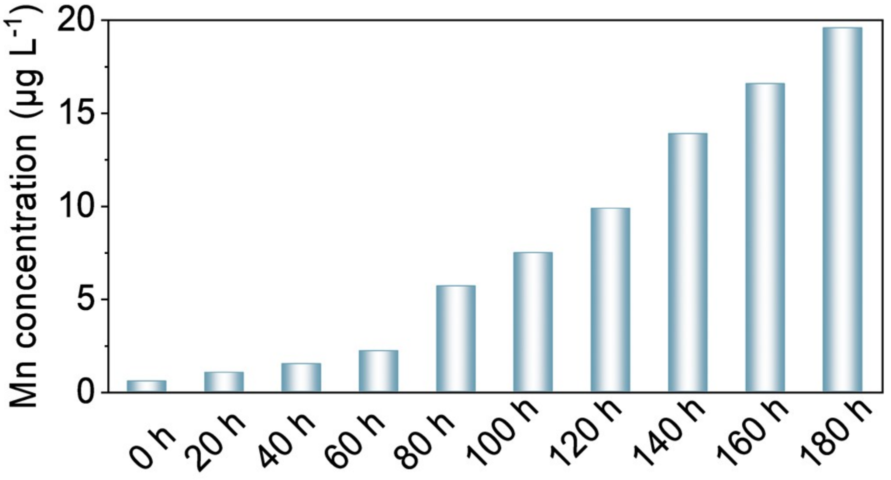

Figure S18. The change in the amount of Mn dissolved in the Mn-CoO/HMCS stability test.

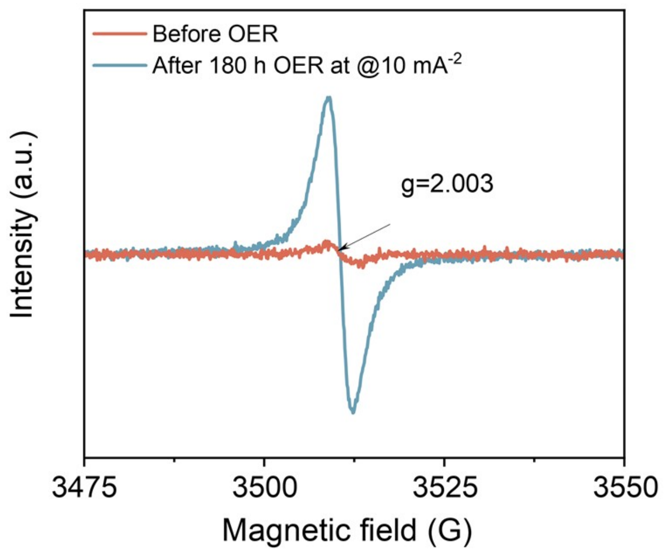

Figure S19. EPR spectra of Mn-CoO/HMCS before and after the stability test.

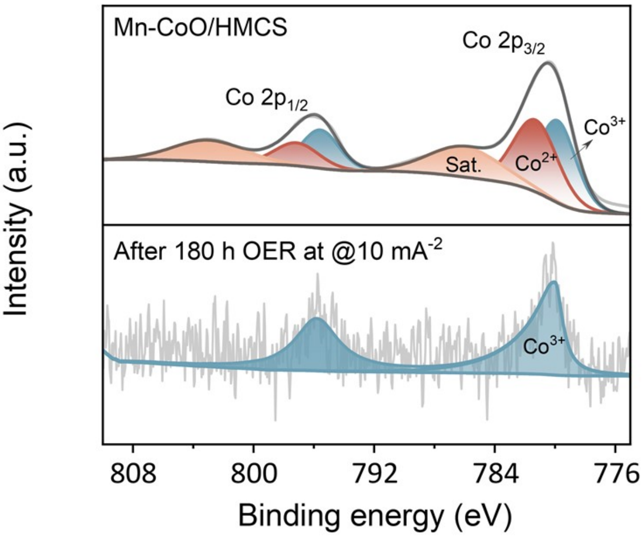

Figure S20. XPS spectra of Co 3d before and after the stability test for Mn-CoO/HMCS.

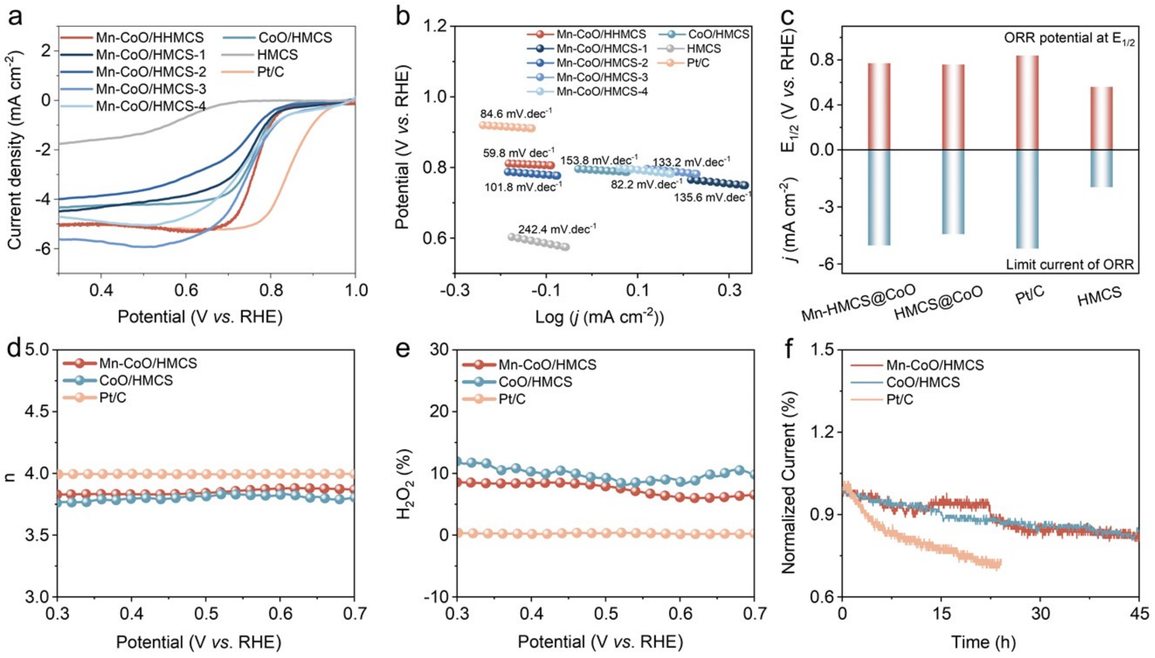

Figure S21. ORR performance: (a) LSV curves of Mn-CoO/HMCS, CoO/HMCS, HMCS, and Pt/C in 0.1 M KOH solution at 1600 rpm, (b) Tafel plots, (c) ORR performance comparison, (d) Corresponding electron transfer number n at different potentials, (e) Percentage of peroxide concerning the total oxygen reduction products. and (f) Comparison of the stability between Mn-CoO/HMCS and CoO/HMCS.

The ORR performance of the prepared catalysts and commercial Pt/C was tested using a rotating ring-disk electrode (RRDE) in 0.1 M KOH solutions. Mn-CoO/HMCS demonstrates the optimal oxygen reduction activity among various manganese doping levels, with a half-wave potential ($E_{1/2}$) of 0.767 volts (Figure S16a). To study the ORR reaction kinetics, Tafel slopes of the catalysts were also assessed (Figure S16b). As expected, Mn-CoO/HMCS displays a smaller Tafel slope compared to other catalysts (59.8 mV dec^{-1}). The half-wave potential and limiting current density are summarized in Figure S16c. Mn-CoO/HMCS shows superior performance compared to CoO/HMCS and HMCS, approaching that of commercial 20% Pt/C. In addition to activity, the ORR pathway and selectivity were also evaluated by rotating ring-disk electrode (RRDE) measurement. The number of electron transfers of 3.83–3.97 on Mn-CoO/HMCS suggests a mechanism close to four-electron pathway for electron transfer (Figure S16d). The yield of H_2O_2 less than 10% is consistent with the four-electron reaction pathway (Figure S16e). Moreover, as the doped manganese content increases, it approaches the four-electron reaction pathway more closely (Figure S17a-b). In the stability testing, due to the confinement effect of the mesoporous structure, both Mn-CoO/HMCS and Co/HMCS exhibit ORR durability that surpasses Pt/C (Figure S16f).

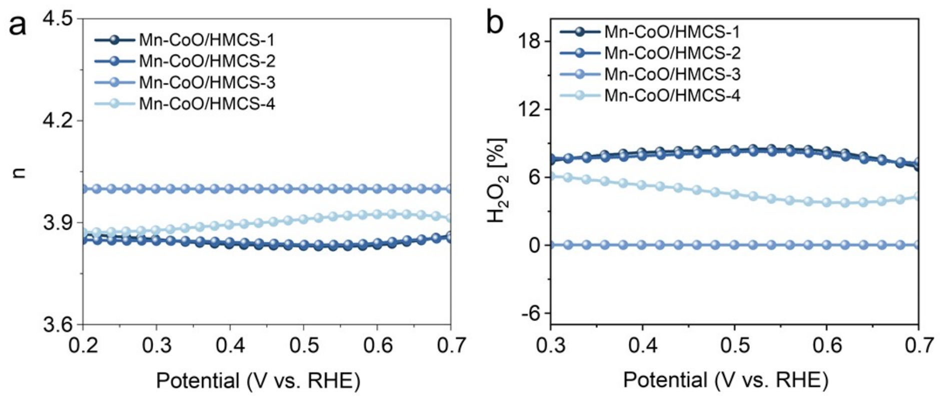

Figure S22. (a) Corresponding electron transfer number n at different potentials, (b) Percentage of peroxide concerning the total oxygen reduction products.

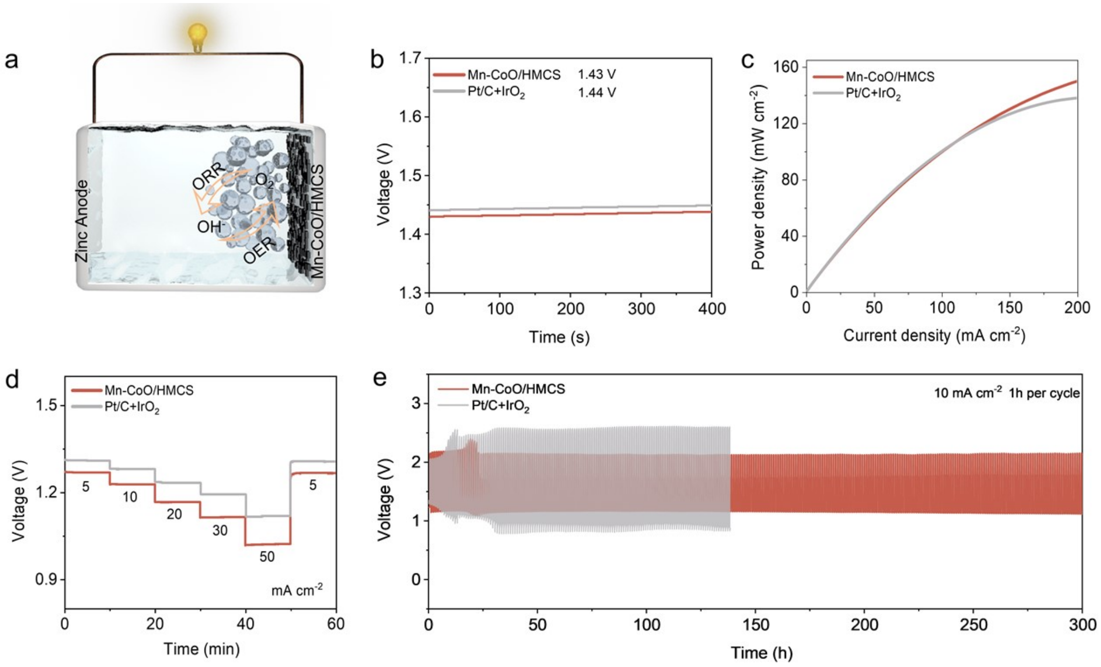

Figure S23. Performance of zinc-air batteries with the Mn-CoO/HMCS and Pt/C+IrO_2 as the catalyst used in an air cathode. (a) Schematic illustration of home-made Zinc–air battery. (b) Open-circuit plots. (c) power density curves. (d) Galvanostatic discharge curves of the zinc-air battery at different current densities. (e) cycle stability plot at a discharge and charge current density of 10 mA cm^{-2}.

The open-circuit voltage (OCV) of 1.43 V is slightly lower than that with 20% Pt/C+IrO_2 catalyst (1.44 V) (Figure S18b). Furthermore, this ZAB exhibits a peak power density of 149.9 mW cm^{-2}, outperforming that of Pt/C-based (137.5 mW cm^{-2}) (Figure S18c). In addition, the constant current discharge voltage of Mn-CoO/HMCS under different current densities (5–50 mA cm^{-2}) is slightly lower than that of Pt/C, showing excellent ORR stability and rate performance (Figure S18d). In the constant current charge and discharge test (Figure S18e), the charge and discharge voltage of the Mn-CoO/HMCS ZAB is unchanged after 300 h continuous operation, which is much better than commercial Pt/C. Its charge-discharge curves were also comparable to those of precious metals (Figure S19). These findings collectively demonstrate that Mn-CoO/HMCS materials exhibit superior performance in zinc-air battery applications.

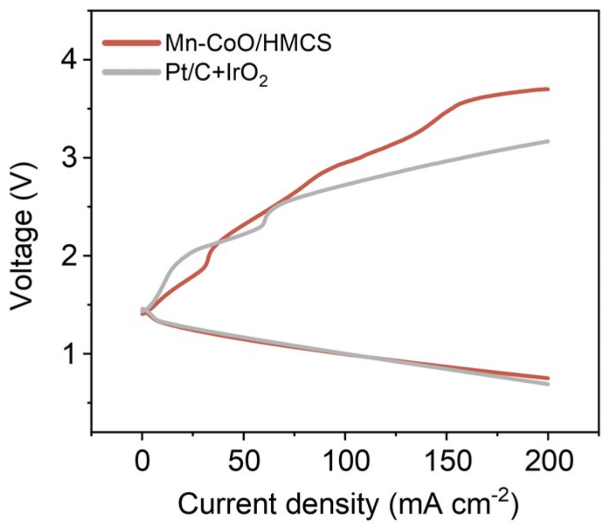

Figure S24. Charging and discharging polarization curves of the as-assembled zinc-air batteries.

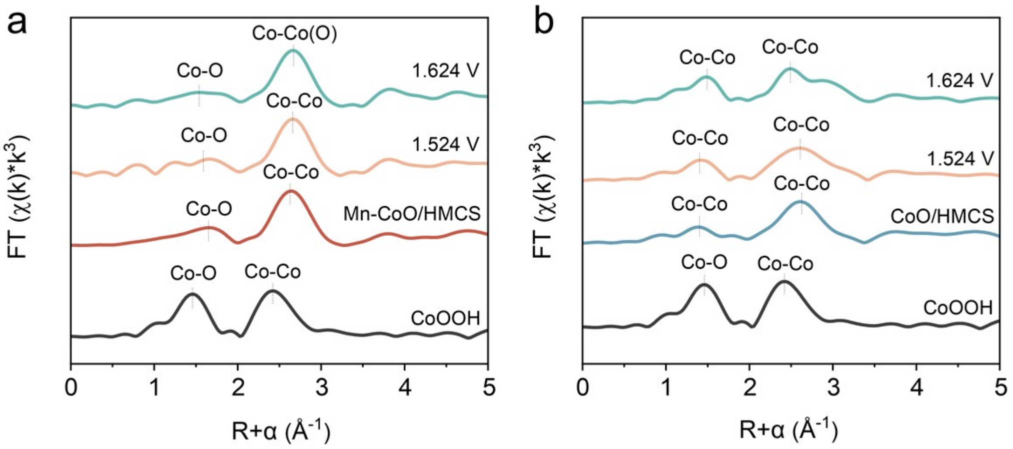

Figure S25. (a) FT-EXAFS spectra of Mn-CoO/HMCS, Mn-CoO/HMCS@1.524 V, Mn-CoO/HMCS@1.624 V, and standard sample CoOOH. (b) FT-EXAFS spectra of CoO/HMCS, CoO/HMCS@1.524 V, CoO/HMCS@1.624 V, and standard sample CoOOH.

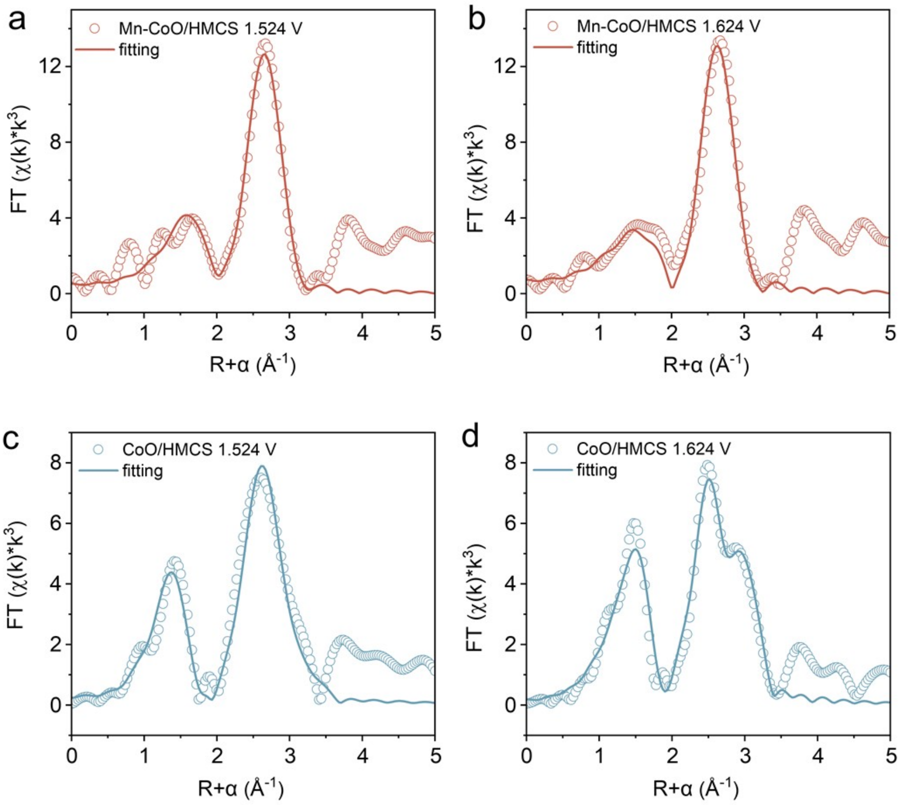

Figure S26. FT-EXAFS fitting results of (a) Mn-CoO/HMCS@1.524 V, (b) Mn-CoO/HMCS@1.624 V, (c) CoO/HMCS@1.524 V, and (d) CoO/HMCS@1.624 V.

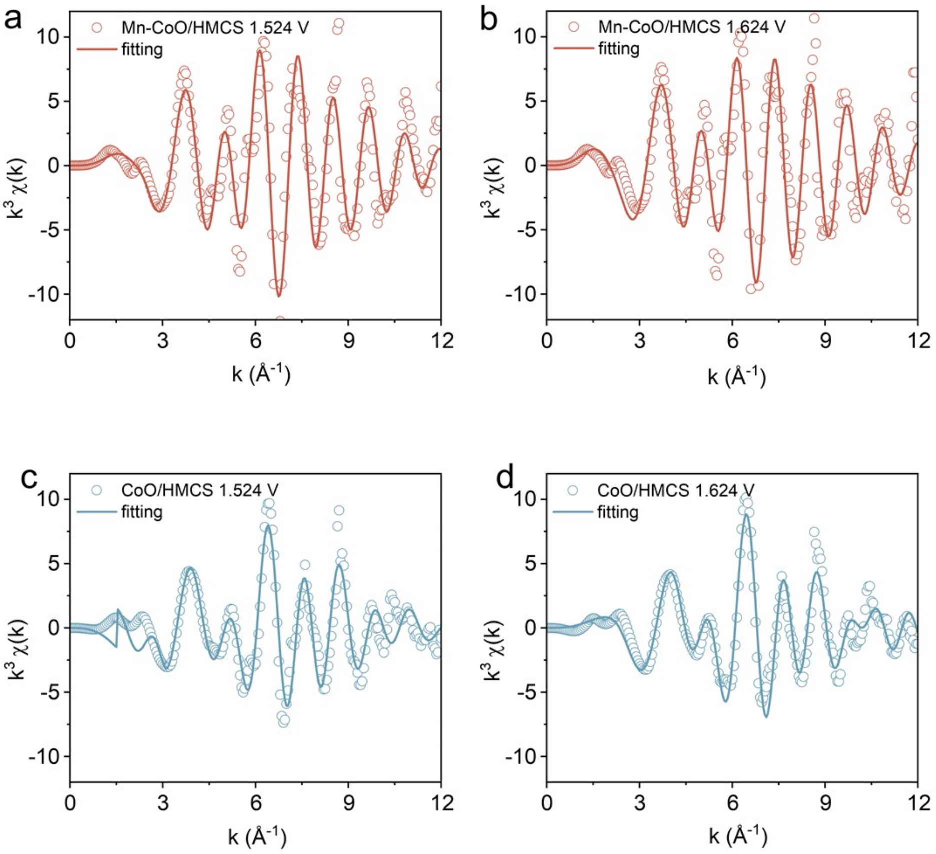

Figure S27. EXAFS fitting curves at K space of Co K-edge for (a) Mn-CoO/HMCS @1.524 V, (b) Mn-CoO/HMCS@1.624 V, (c) CoO/HMCS@1.524 V, and (d) Mn-CoO/HMCS@1.624 V.

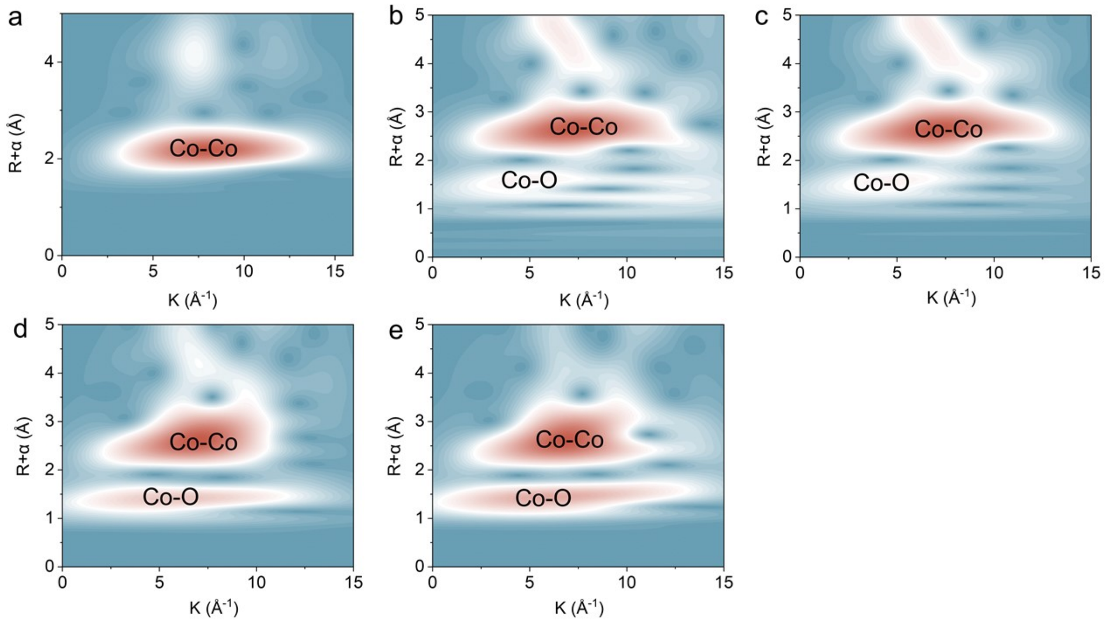

Figure S28. WT-EXAFS contour plots of (a) Co foil, (b) Mn-CoO/HMCS@1.524 V, (c) Mn-CoO/HMCS@1.624 V, (d) CoO/HMCS@1.524 V, and (e) CoO/HMCS@1.624 V.

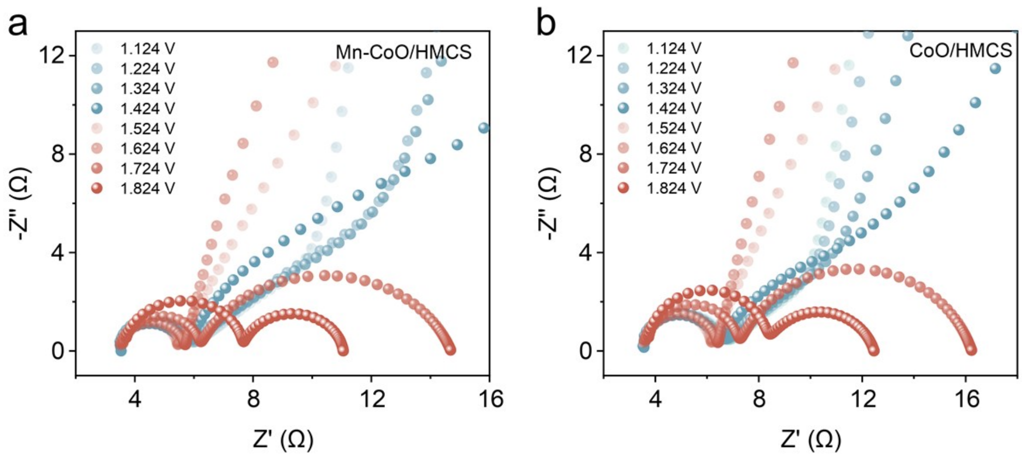

Figure S29. (a) Nyquist plots of Mn-CoO/HMCS for OER in different potentials. (b) Nyquist plots of CoO/HMCS for OER in different potentials.

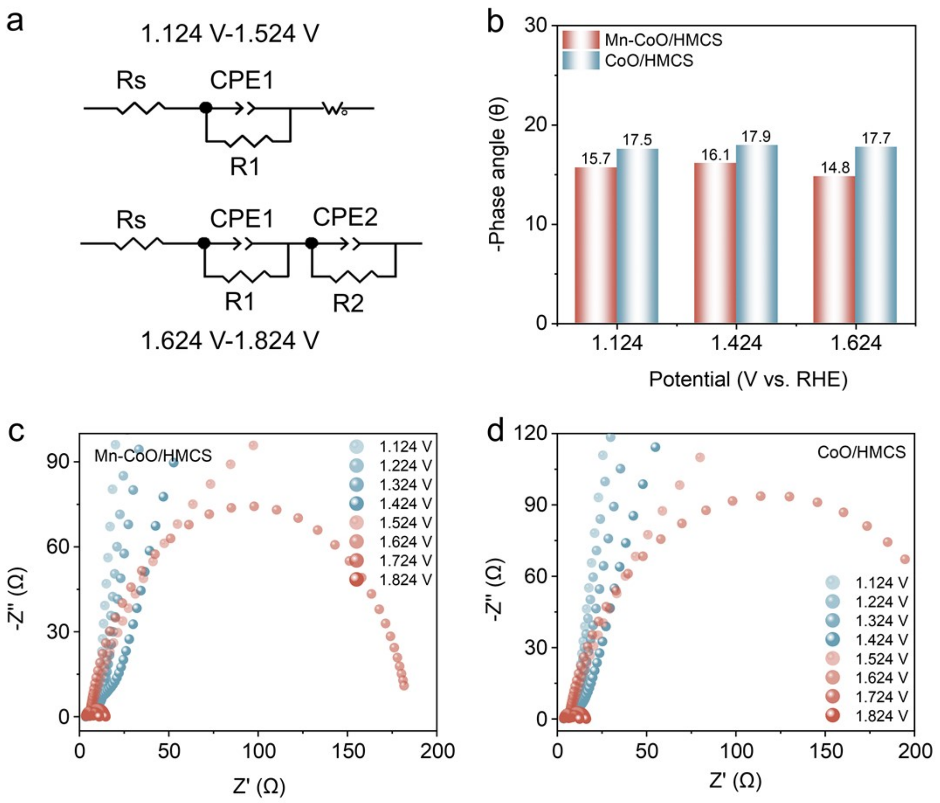

Figure S30. (a) Equivalent circuit models for OER. (b) The phase angle of Mn-CoO/HMCS and CoO/HMCS at high frequency region. (c) Nyquist plots of Mn-CoO/HMCS for OER in different potentials. (d) Nyquist plots of CoO/HMCS for OER in different potentials.

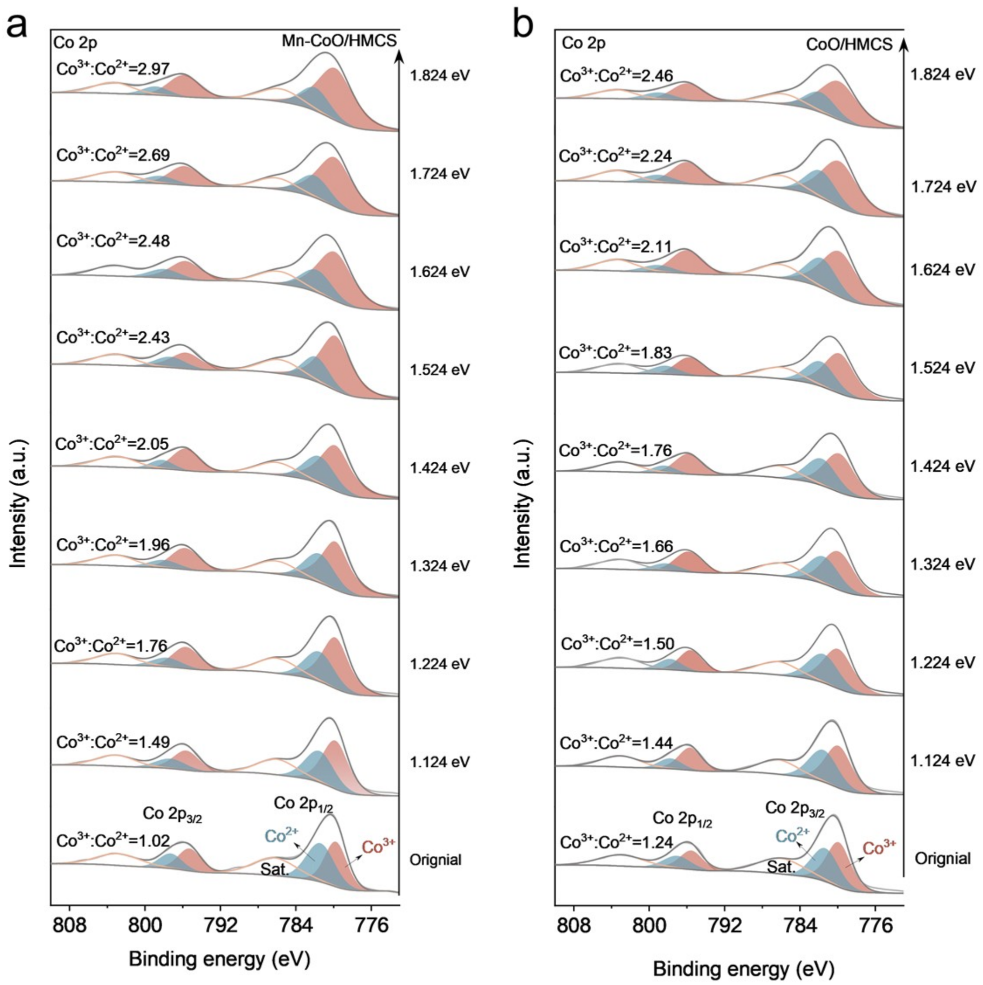

Figure S31. (a) In situ high-resolution XPS spectra of Co 2p for Mn-CoO/HMCS. (b) In situ high-resolution XPS spectra of Co 2p for CoO/HMCS.

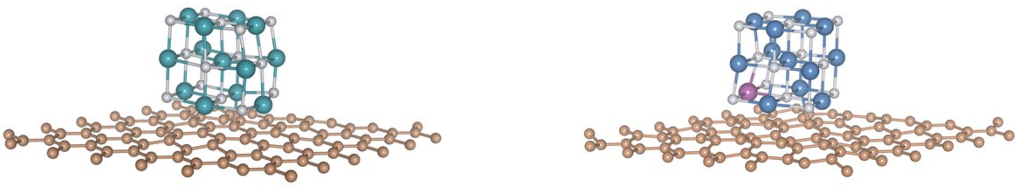

CoO/HMCS Mn-CoO/HMCS

Figure S32. Schematic structure model of CoO/HMCS and Mn-CoO/HMCS.

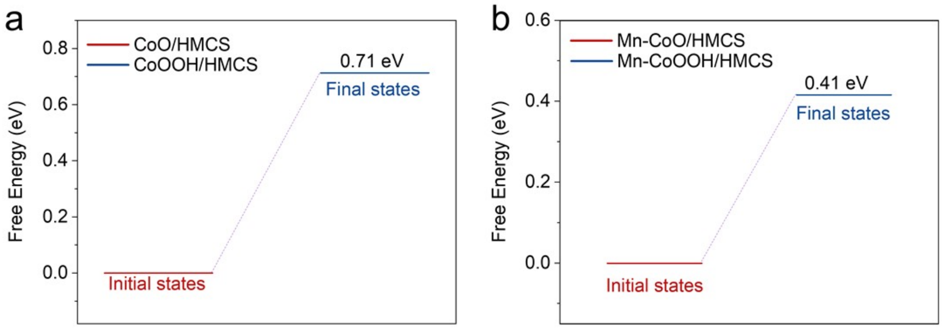

Figure S33. The formation energy for the corresponding formation of CoOOH in (a) Mn-CoO/HMCS and (b) CoO/HMCS.

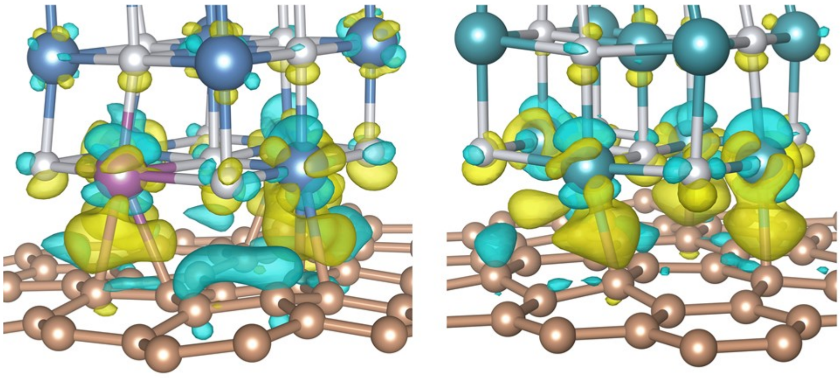

Figure S34. The adsorption charge density on the catalyst models of Mn-CoO/HMCS and CoO/HMCS.

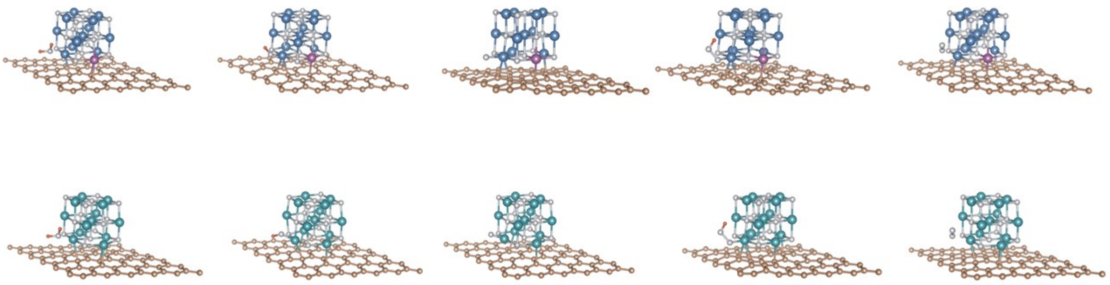

*   *OH   *O   *OOH O_2

Figure S35. Reaction energy diagram of water oxidation on Mn-CoO/HMCS and CoO/HMCS models at different stages of the OER reaction.

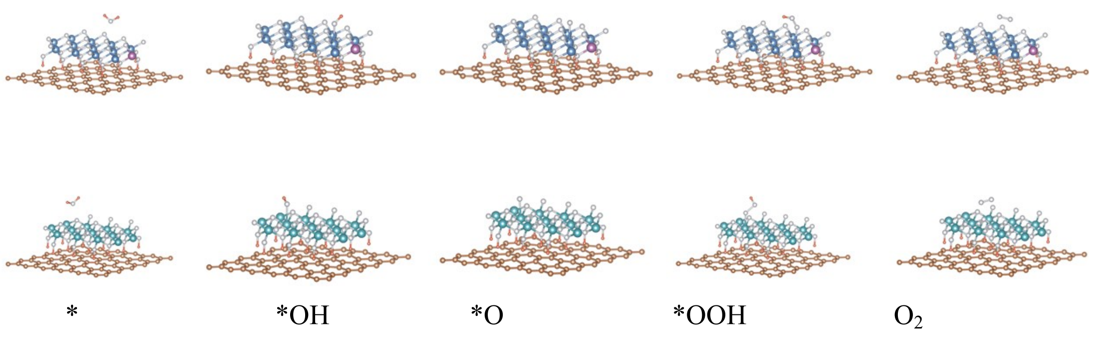

Figure S36. Reaction energy diagram of water oxidation on Mn-CoOOH/HMCS and CoOOH/HMCS models at different stages of the OER reaction.

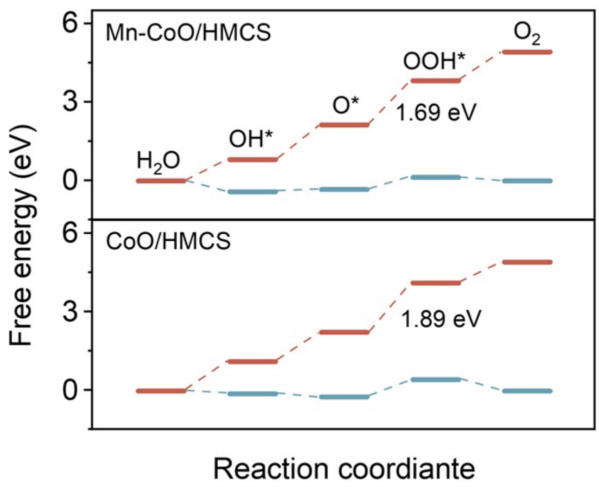

Figure S37. Gibbs free energy diagrams of Mn-CoO/HMCS and CoO/HMCS under the bias potential of 0 V and 1.23 V vs reversible hydrogen electrode (RHE).

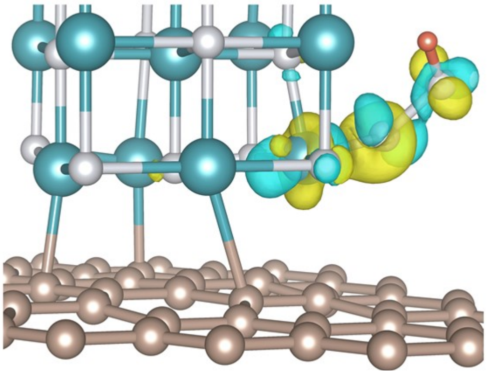

Figure S38. The charge density of *OOH adsorption on the CoO/HMCS catalyst model.

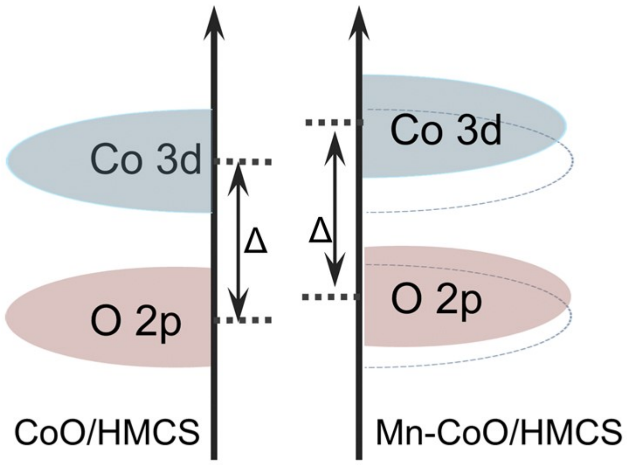

Figure S39. Schematic diagram for the d-band center change of the sample.

Table S1. Performance comparison of Mn-CoO/HMCS with other previously reported transition metal catalysts at 1.0 M KOH

Catalysis	Electrolyte	(mV vs. RHE) @ 10mAcm^{-2}	Tafel slope (mV/dec)	Ref.
**Mn-CoO/HMCS**	**1.0 M KOH**	**360**	**53.7**	**This work**
Zn-Co_3O_4	1.0 M KOH	370	63	7
PCNSs	1.0 M KOH	368	59	8
MFO	1.0 M KOH	396	80	9
Co-C_3N_4/CNT	1.0 M KOH	380	68	10
CoFe-N-C	1.0 M KOH	360	68	11
Co-NCNT	1.0 M KOH	370	56	12
CoP	1.0 M KOH	400	57	13
Co@NC-3/1	1.0 M KOH	370	90	14
Co–NHGF	1.0 M KOH	402	80	15
CoSAs@CNTs	1.0 M KOH	410	85	16
Fe-N4 SAs/NPC	1.0 M KOH	430	95	17
FeCo-DACs/NC	1.0 M KOH	370	82.7	18
CG-CoO	1.0 M KOH	420	75	19
Co_9S_8@MoS_2/CNFs	1.0 M KOH	430	61	20
Co/N CCPC-3	1.0 M KOH	401	103	21
LSCO10	1.0 M KOH	381	75	22
CoS	1.0 M KOH	347	81	23
$NiFe_2O_4$-H_2	1.0 M KOH	389	64	24
Fe/mCo_3O_4	1.0 M KOH	380	60	25
Co_3O_4 nanotubes	1.0 M KOH	390	76	26

Table S2. Composition of all samples detected by ICP-AEC measurement

Samples	Co wt.%
CoO/HMCS	20.036
Mn-CoO/HMCS	16.662

Table S3. ICP-MS results of the Mn-CoO/HMCS immersed electrolytes with increasing soaking time

Immersing time	Co [μg L^{-1}]	Mn [μg L^{-1}]
Pristine	0.8005	0.5515
20 hours	0.8903	1.0231
40 hours	1.4639	1.4864
60 hours	1.4979	2.1838
80 hours	1.5032	5.6701
100 hours	1.6381	7.4650
120 hours	1.6417	9.8634
140 hours	1.6790	13.8837
160 hours	1.9566	16.5735
180 hours	2.1819	19.5878

References

1. X. K. Wan, H. B. Wu, B. Y. Guan, D. Luan and X. W. Lou, *Adv. Mater.*, 2019, **32**, 1901349.
2. G. Kresse and J. Furthmüller, *PhRvB*, 1996, **54**, 11169.
3. G. Kresse and J. Furthmiiller, *Comput. Mater. Sci*, 1996, **6**, 15.
4. J. P. Perdew, K. Burke and M. Ernzerhof, *Phys. Rev. Lett.*, 1996, **77**, 3865.
5. G. Kresse and D. Joubert, *PhRvB*, 1999, **58**, 1758.
6. P. E. Blöchl, *PhRvB*, 1994, **50**, 17953-17979.
7. Y. Yang, L. Xu, W. Wang, R. Han, J. Ma, M. Yao, S. Geng and F. Liu, *JMatS*, 2023, **58**, 5234-5243.
8. Z. Li, X.-Y. Yu and U. Paik, *J. Power Sources*, 2016, **310**, 41-46.
9. A. Maurya and M. Yadav, *J. Alloys Compd.*, 2023, **956**, 170208.
10. Y. Zheng, Y. Jiao, Y. Zhu, Q. Cai, A. Vasileff, L. H. Li, Y. Han, Y. Chen and S.-Z. Qiao, *J. Am. Chem. Soc.*, 2017, **139**, 3336-3339.
11. X. Zhou, J. Gao, Y. Hu, Z. Jin, K. Hu, K. M. Reddy, Q. Yuan, X. Lin and H.-J. Qiu, *Nano Lett.*, 2022, **22**, 3392-3399.
12. J. Cong, C. Li, T. Zhao, J. Wu, R. Zhang, W. Ren, S. Wang, J. Gao, Y. Liu and J. Yao, *J. Solid State Chem.*, 2017, **253**, 227-230.
13. M. Liu and J. Li, *ACS Appl. Mater. Interfaces*, 2016, **8**, 2158-2165.
14. Y. Li, B. Jia, Y. Fan, K. Zhu, G. Li and C. Y. Su, *Adv. Energy Mater.*, 2017, **8**, 1702048.
15. H. Fei, J. Dong, Y. Feng, C. S. Allen, C. Wan, B. Volosskiy, M. Li, Z. Zhao, Y. Wang, H. Sun, P. An, W. Chen, Z. Guo, C. Lee, D. Chen, I. Shakir, M. Liu, T. Hu, Y. Li, A. I. Kirkland, X. Duan and Y. Huang, *Nat. Catal.*, 2018, **1**, 63-72.
16. S. Dilpazir, H. He, Z. Li, M. Wang, P. Lu, R. Liu, Z. Xie, D. Gao and G. Zhang, *ACS Appl. Energy Mater.*, 2018, **1**, 3283-3291.
17. Y. Pan, S. Liu, K. Sun, X. Chen, B. Wang, K. Wu, X. Cao, W. C. Cheong, R. Shen, A. Han, Z. Chen, L. Zheng, J. Luo, Y. Lin, Y. Liu, D. Wang, Q. Peng, Q. Zhang, C. Chen and Y. Li, *Angew. Chem. Int. Ed.*, 2018, **57**, 8614-8618.
18. M. Liu, N. Li, S. Cao, X. Wang, X. Lu, L. Kong, Y. Xu and X. H. Bu, *Adv. Mater.*, 2022, **34**, 2107421.
19. S. Mao, Z. Wen, T. Huang, Y. Hou and J. Chen, *Energy Environ. Sci.*, 2014, **7**, 609-616.
20. H. Zhu, J. Zhang, R. Yanzhang, M. Du, Q. Wang, G. Gao, J. Wu, G. Wu, M. Zhang, B. Liu, J. Yao and X. Zhang, *Adv. Mater.*, 2015, **27**, 4752-4759.
21. T. Wang, Y. He, Y. Liu, F. Guo, X. Li, H. Chen, H. Li and Z. Lin, *Nano Energy*, 2021, **79**, 105487.
22. X. Li, Y. Bai and Z. Cheng, *Adv. Sci.*, 2021, **8**, 2101000.
23. H. Xu, J. Cao, C. Shan, B. Wang, P. Xi, W. Liu and Y. Tang, *Angew. Chem. Int. Ed.*, 2018, **57**, 8654-8658.
24. D. Lim, H. Kong, N. Kim, C. Lim, W. S. Ahn and S. H. Baeck, *ChemNanoMat*, 2019, **5**, 1296-1302.
25. C. Xiao, X. Lu and C. Zhao, *Chem. Commun.*, 2014, **50**, 10122--10125.
26. H. Wang, S. Zhuo, Y. Liang, X. Han and B. Zhang, *Angew. Chem. Int. Ed.*, 2016, **55**, 9055-9059.
